# Sickle cell disease and maternity care: A qualitative synthesis of women’s and health providers experiences

**DOI:** 10.1371/journal.pone.0352992

**Published:** 2026-07-13

**Authors:** Kenneth Finlayson, Gill Moncrieff, Cath Harris, Soo Downe

**Affiliations:** 1 School of Nursing and Midwifery, University of Central Lancashire, Preston, United Kingdom; 2 Nursing Midwifery and Allied Health Professions Research Unit, University of Stirling, Scotland, United Kingdom; 3 Applied Health Research Hub, University of Central Lancashire, Preston, United Kingdom; PLOS: Public Library of Science, UNITED KINGDOM OF GREAT BRITAIN AND NORTHERN IRELAND

## Abstract

**Introduction:**

Sickle Cell Disease (SCD) is one of the most prevalent genetic diseases in the world. For women with SCD or those with Sickle Cell Trait (SCT) reproductive choices not only require consideration of genetic inheritance but also the exacerbation of pregnancy induced SCD symptoms and potential birth complications. To explore these issues a qualitative synthesis was conducted incorporating the views of women with SCD or SCT as well as healthcare professionals working with these women in maternity settings.

**Methods:**

Database searches were performed in MEDLINE, Embase, MIDIRS, CINAHL Ultimate, PsycINFO, Social Sciences Citation Index, Global Index Medicus and CNKI, as well as reference lists of included studies published January 2000–October 2024. Studies reporting qualitative data from women with SCD or SCT and health professionals providing care to these women during the maternity phase were included.

**Data Collection and Analysis:**

Author findings were extracted, coded and synthesised using techniques derived from thematic synthesis. Confidence in the quality, coherence, relevance and adequacy of data underpinning the resulting findings was assessed using GRADE-CERQual.

**Results:**

Eleven studies from four different countries (UK, Brazil, France and Uganda) were identified including eight focusing on the views of women, two reporting on the views of health professionals and one exploring both. From these four analytical themes were generated: *Beyond the routine of antenatal screening; Myths, misunderstandings and submissiveness; Fear and uncertainty in the face of adversity; The importance of familial and organizational support*. Confidence in the results was moderate to good.

**Conclusion:**

The synthesis suggests that the intersection of SCD and maternity care is beset with a lack of understanding and inadequate health system support. In some contexts, women endure stigmatization, discrimination and poor quality care. Public awareness campaigns and educational initiatives, particularly amongst healthcare professionals, are urgently required to address these issues.

## Introduction

Sickle cell disease (SCD) is an umbrella term that incorporates several inherited diseases (including sickle cell anaemia (SCA), HbSC and HbSβ-thalassaemia) characterized by genetic mutations affecting the haemoglobin molecule [1]. As a consequence of these mutations red blood cells develop a characteristic sickle shape which can cause blockages in the blood vessels of people with SCD. These blockages may lead to recurrent and painful crises as well as organ and/or tissue damage [2]. SCD is also associated with severe anaemia, cerebrovascular disease, and premature mortality [3]. In addition, many infectious diseases, such as pneumococcal disease, are more dangerous to people with SCD [4] and its impact on quality of life can lead to a higher incidence of depression [5].

Recent reports suggest there are approximately 6.4 million people living with SCD globally, and from 2010 to 2050, the number of children born with SCD is expected to increase by around 30% [6]. Although SCD is more prevalent in Sub-Saharan Africa, the Middle East and parts of Southern Asia, regular waves of migration from these regions have led to an increased incidence of the condition in North America and Western Europe [7]. Over the last several decades the prognosis for people born with SCD has improved, particularly in High-Income Countries (HICs) where comprehensive screening programmes, effective treatments, specialist services and better management of the condition has extended life expectancy [8,9]. However, in many Low and Middle-Income Countries (LMICs) the impact of SCD is stark with up to 90% of infants born with the condition dying before they reach the age of five [10].

Due to the risks associated with transferring the sickle cell gene mutation on to their children reproductive decision-making can be a complex process for women with SCD. Their choice of partner, as well as views on contraception and antenatal screening all require careful consideration. For some women, in certain contexts, the option to terminate an affected foetus, may lead to particularly difficult and distressing decision-making. In addition, pregnancy can exacerbate SCD related symptoms including vaso-occlusive crises, severe anaemia, acute chest syndrome and extreme joint pain [11,12]. Pregnancy complications can also lead to pre-eclampsia, fetal growth restriction, stillbirth, and preterm delivery in women with SCD [13].

Faced with these challenges it is important that women who have the disease or carry the affected gene, known as Sickle Cell Trait (SCT), are given relevant information and are supported by healthcare providers about their reproductive choices. However, evidence from survey-based studies suggest that understanding of the condition and its impact on reproductive health is poor amongst women with SCD or SCT [14–16]. Knowledge about SCD and SCT may also be lacking amongst maternity providers [17–19].

In addition, some studies suggest that women with SCD or SCT face stigmatization from healthcare professionals or are given false or misleading information about their reproductive choices [20,21]. Given these concerns it is vital that the views and experiences of women with SCD or SCT (as well as the health professionals providing care to them) are explored in more detail to fully understand the underlying challenges associated with maternity care provision for this population of women. Qualitative studies conducted in a maternity context are likely to generate insights in this regard, so the review sought to:-

a) Identify and synthesize qualitative studies reporting on the views and experiences of women with SCD or SCT during their encounters with maternity providers.
b) Identify and synthesize qualitative studies reporting on the views and experiences of maternity providers offering care to women with SCD or SCT.

This review forms part of a programme of work conducted by the World Health Organization (WHO) seeking to explore the impact of Non-Communicable Diseases (NCDs) in maternity care with a view to developing guidelines in this area. The WHO have prioritized five NCDs, namely diabetes, cardiovascular diseases, respiratory diseases, haemoglobinopathies and mental health & substance abuse. As part of the haemoglobinopathies review, this article explores the impressions and experiences of women with SCD or SCT during their maternity journey, as well as the experiences of maternity providers offering care to women with this disease.

## Methods

A qualitative systematic review was conducted and reported in accordance with PRISMA guidelines. Study assessment included the use of a validated quality appraisal tool [22]. Thematic synthesis techniques [23] were used for analysis and synthesis and the GRADE-CERQual tool [24] was applied to the review findings to assess confidence in each finding. The protocol for the full WHO study programme was published on PROSPERO (Registration Number CRD42023452405) in August 2023.

### Criteria for Inclusion

The topic of interest was studies exploring the experiences of women with SCD or SCT during their encounters with antenatal, intrapartum or postnatal care (up to 6 weeks post discharge). To provide further context studies reporting on the views and experiences of health professionals providing maternity care (antenatal, intrapartum or postnatal) to women with SCD or SCT were also included. Qualitative studies and mixed methods studies that utilized a qualitative component, either for design (i.e., ethnography, phenomenology), data collection (i.e., focus groups, interviews, observations, diaries, oral histories), or analysis (i.e., thematic analysis, framework approach, grounded theory) were all included. To qualify for inclusion studies had to report first-hand accounts of women with SCD or SCT or health professionals providing maternity care to women with SCD or SCT in recognized health facilities (primary, secondary or specialist SCD centres). Studies reporting on haemoglobinopathy screening alongside the general population were also included provided there was sufficient data from women diagnosed with SCT. Studies published before 2000 were excluded to ensure that the data reflected the views of the most recent generation of women of childbearing age. Studies reporting on the experiences of women with SCD or SCT outside of maternity care (i.e., experiences of general healthcare) were also excluded as were studies reporting on experiences of non-routine diagnostic tests, e.g., non-invasive prenatal testing (NIPD). A summary of the inclusion and exclusion criteria is shown in Table 1 below.

**Table 1:** Summary of inclusion and exclusion criteria.

Inclusion Criteria	Exclusion Criteria
• Qualitative or mixed method studies reporting on the experiences of women with SCD or SCT during the maternity phase • Qualitative or mixed method studies reporting on the experiences of health professionals providing maternity care to women with SCD or SCT • Qualitative or mixed method studies published between Jan 2000 – Oct 2024.	• Quantitative studies • Qualitative or mixed method studies reporting on the experiences of women with SCD or SCT outside of the maternity phase, e.g., pre-conception care or general healthcare. • Qualitative or mixed method studies reporting on the experiences of health professionals providing care to women with SCD or SCT outside of the maternity phase • Qualitative or mixed method studies published before 2000. • Qualitative or mixed method studies reporting on women’s experiences of foetal or neonatal testing. • Qualitative or mixed method studies reporting on the experiences of women with other genetic conditions (e.g.,thalassemia) during the maternity phase • Qualitative or mixed method studies reporting on the experiences of health professionals providing maternity care to women with other genetic conditions.

### Search strategy

A search strategy was developed in conjunction with a university information specialist (CH) utilizing a PEO (Population, Exposure, Outcome) structure with the addition of search terms to identify qualitative studies. Systematic searches were carried out on 18th July 2023 and updated on 31^st^ October 2024 utilizing the following databases, Ovid MEDLINE, Ovid Embase, Ovid MIDIRS, CINAHL Ultimate (EBSCOhost), PsycINFO (EBSCOhost), Social Sciences Citation Index (via Web of Science), Global Index Medicus (all indexes) and CNKI (China National Knowledge Institute). Searches were limited by date to publications from 2000 onwards. No language restrictions were imposed, although all searches were conducted in English. Search strategies for each database are shown in the S1 Appendix.

### Study selection

After removing duplicates in EndNote records were uploaded into Rayyan and, where possible, irrelevant records were removed based on title. Two members of the review team (KF, GM) independently assessed 50% of the abstracts each to determine eligibility against the *a priori* inclusion/exclusion criteria. Twenty percent of the total number of abstracts were screened in duplicate to ensure consistency between the reviewers and, in the event of a discrepancy, agreement on inclusion was reached by consensus. Following the removal of unrelated articles the full texts of potentially relevant articles were obtained and independently assessed for eligibility by KF and GM. References for books and book chapters identified from the database searches were acquired through the University library and assessed for eligibility, i.e., peer reviewed, reporting relevant, empirical, qualitative data. The reference lists of included articles were examined at this stage to check for further studies not identified in the searches. KF and GM selected relevant articles for inclusion with adjudication by a third author (SD) in the event of any disagreement. For studies published in languages other than English the full texts were translated into English using freely available online software (Google Translate) [25] and the decision to include or exclude was taken by consensus between GM and KF.

### Quality appraisal

Included studies were appraised using an instrument developed by Walsh and Downe [22] and modified by Downe et al [26]. Studies were rated against 11 pre-defined criteria, and then allocated a score from A–D, where A represented a study with no, or few flaws, with high credibility, transferability, dependability and confirmability; B, a study with some flaws, unlikely to affect the credibility, transferability, dependability and/or confirmability of the study; C, a study with some flaws that may affect the credibility, transferability, dependability and/or confirmability of the study; and D, a study with significant flaws that are very likely to affect the credibility, transferability, dependability and/or confirmability of the study. Each study was appraised by two authors independently (KF, GM) and then cross-checked by the same authors to ensure consistency. Any studies where there were scoring discrepancies of more than a grade were referred to another author (SD) for moderation. Studies scoring C or higher were included in the final analysis.

### Data extraction and analysis

An Excel spreadsheet was used to record pertinent details for each included study. i.e., author(s); date of publication; language; country where study conducted; resource setting; study population (women or health professional); phase of maternity care (antenatal, intrapartum, postnatal); study type and participant details. Details of the quality appraisal score for each study were also recorded on this spreadsheet. The data extraction was carried out by KF and GM between the dates of 14^th^ and 29^th^ November 2023.

In a process aligned with thematic synthesis [23] two review authors (KF & GM) worked together to develop codes from the author findings and quote material in the included papers. In studies where the author findings included data from participants not relevant to the research question, i.e., participants who did not have SCD or SCT or had thalassemia rather than SCD, efforts were made to code and extract data from pertinent sections of the text. Using an iterative process the same two authors (KF, GM) then synthesized these codes into descriptive themes (review findings) and reached agreement on the analytical themes following input and discussion from a third author (SD). KF conducted GRADE-CERQual [24] assessments of the descriptive themes (review findings) and the final grade was reached by consensus following a cross-check of each assessment by GM. Any grade disagreements were referred to SD for moderation. GRADE-CERQual assesses the methodological limitations and relevance of the studies contributing to a review finding, the coherence of the review finding, and the adequacy of data supporting a review finding. Based on these criteria, review findings were graded for confidence using a classification system ranging from ‘high’ to ‘moderate’ to ‘low’ to ‘very low’ [24]. Following GRADE-CERQual assessment the review findings were grouped into higher order, analytical themes and agreed by consensus amongst the authors. The data extraction, analysis, synthesis and CERQual grading stages are illustrated in the S2 Appendix.

## Results

**Fig 1:**
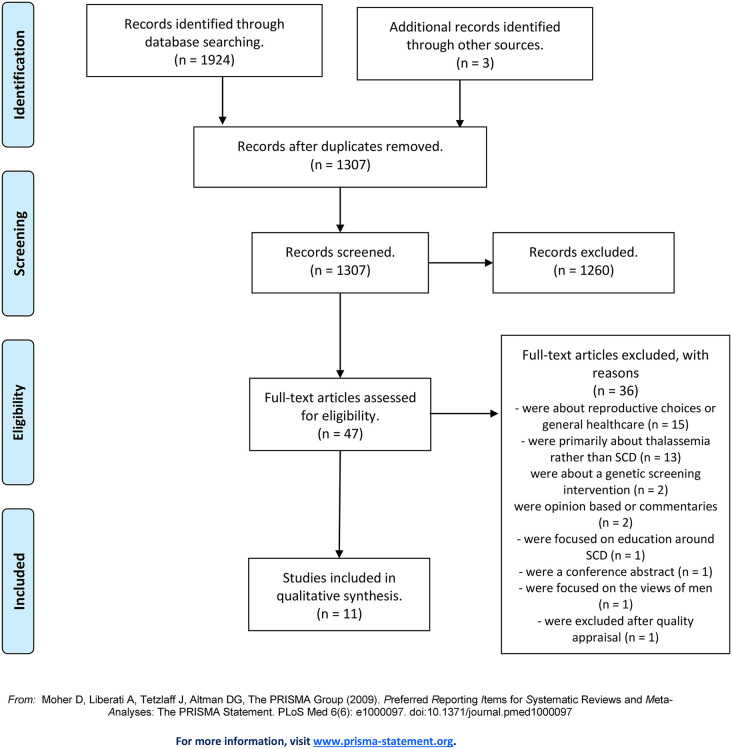
PRISMA Flow Diagram.

The systematic database searches yielded 1924 results and an additional three articles were obtained following reference checking. A list of all articles (1927 in total) retrieved through the searches is shown in the S3 Appendix. Six hundred and twenty articles were excluded after deduplication leaving 1307 for screening. Six hundred and sixty-two of these were excluded by title and a further 598 excluded by abstract leaving 47 for full-text review. Of these, 35 were excluded because they failed to fulfil the inclusion criteria so 12 studies were taken forward for quality appraisal. One study was excluded at this stage [27] after scoring D because of very limited details about data collection methods, analysis and theme development. A full list of articles considered for inclusion incorporating the reason(s) for exclusion is shown in the S4 appendix. The remaining 11 papers were included in the final synthesis. See Fig 1 for the PRISMA flow diagram illustrating the study selection process and the S5 Appendix for the PRISMA Checklist.

The 11 studies were conducted in four different countries including six from the UK, three from Brazil and one each from France and Uganda. They were generally of moderate to good quality with one scoring B + , four scoring B, four scoring C+ and two scoring C. All studies were available in English so we did not need to use Google Translate software. Eight studies reported on women’s experiences, two reported on the views of health professionals and one incorporated both women and health professional perspectives. Six of the studies were focused on experiences of antenatal screening for haemoglobinopathies (including SCT) with the remainder exploring various aspects of the maternity journey for women with SCD. For the six studies focused on screening some of the findings included data from participants relating to thalassemia and some incorporated data from women who had received a negative SCT screening result. In all of these cases pertinent data was extracted from the population of interest by only coding relevant text and ignoring inappropriate quotes. The characteristics of the included studies are shown in Table 2.

**Table 2:** Characteristics of included studies.

Author, Date of Publication & Title	Country	Resource	Study Design	Study Aims	Participants	Quality Rating
Cox FEM & Beauquier-Maccotta, 2014 [28] B. *Maternal representations during a pathological pregnancy: the case of sickle cell disease.*	France	High	Qualitative, exploratory, and descriptive using individual semi-structured interviews	To explore experiences of pregnancy for women with sickle cell anaemia	5 pregnant women with SCD originally from The French Antilles and Africa	C
Dyson SM, 2005 [29]. *Ethnicity and screening for sickle cell and thalassemia: lessons for practice from the voices of experience [Book]*	UK	High	Qualitative, exploratory, and descriptive using individual semi-structured interviews	To explore the views of nurse counsellors providing support to women undergoing haemoglobinopathy screening in the UK	27 haemoglobinopathy nurse counsellors	B
Dyson SM, Cochran F, Culley L. et al, 2007 [30]. *Ethnicity questions and antenatal screening for sickle cell/thalassaemia (EQUANS) in England: Observation and interview study*	UK	High	Based on an ethnographic approach incorporating observations and semi-structured interviews	To describe understandings that mothers and midwives have of ethnicity, and to explore barriers to the successful implementation of an ethnicity screening question for sickle cell/thalassaemia	Observation of 121 antenatal booking visits with a focus on ethnicity screening questions plus interviews with 111 mothers and 115 interviews with 61 different midwives	C+
Locock L, Kai J, 2008 [31]. *Parents’ experiences of universal screening for haemoglobin disorders: implications for practice in a new genetics era*	UK	High	Qualitative and exploratory using in depth narrative interviews	To explore the experiences of parents offered new universal screening for haemoglobin disorders in order to inform clinical practice	39 people including 8 couples (30 women and 9 men). 30 had experience of gene-carrier identification through antenatal screening. Ethnicity described as African/African – Caribbean (15); Indian subcontinent (10); European (8); Far East (3); Mixed (3)	B
Santos ACC, Cordeiro RC, ASG Xavier, et al. 2012 [32]. *Feelings Of women with sickle cell anaemia with regard to reproductive experiences*	Brazil	Upper Middle	Qualitative, exploratory, and descriptive using individual semi-structured interviews	To describe the feelings experienced by women with sickle cell anaemia with regard to their reproductive experiences	25 women with Sickle Cell Anaemia reflecting on their pregnancies. 56% identified as black; 44% as brown.	C
Silva UB, Ferreira SL, Cordeiro RC, et al, 2021 [33]. *Experiences of women with sickle cell disease who experienced pregnancy losses*.	Brazil	Upper-Middle	Qualitative, exploratory, and descriptive using individual semi-structured interviews	To understand the experiences of women with SCD in relation to pregnancy losses due to spontaneous and stillborn abortion	20 women with SCD who had experienced pregnancy loss. 65% identified as black and 70% had experienced more than 2 pregnancies	C+
Tsianakas V, Calnan M, Atkin K. et al. 2010 [34]. *Offering antenatal sickle cell and thalassaemia screening to pregnant women in primary care: a qualitative study of GPs’ experiences*	UK	High	Qualitative and descriptive using individual semi-structured interviews	To assess the feasibility of offering antenatal haemoglobinopathy screening in primary care at the time of pregnancy confirmation	34 GPs (offering screening for SC&T when pregnancy is first confirmed)	C+
Tsianakas V, Atkin K, Calnan MW. Et al. 2011 [35] *Offering antenatal sickle cell and thalassaemia screening to pregnant women in primary care: a qualitative study of women’s experiences and expectations of participation.*	UK	High	Qualitative and descriptive using individual in-depth interviews	To explore women’s acceptability of, and preferences expressed during, antenatal haemoglobinopathy screening within a multi-cultural context	21 ethnically diverse women (being offered screening for SC&T at first booking with GP). Ethnicity described as Black African (3); Black Caribbean (5); White (7); South Asian (4); Chinese (1); White European (1)	B
Tumwesige K, Imelda N, Herbert K. et al. 2019 [36]. *Lived experiences of pregnancy among women with sickle cell disease receiving care at Mulago hospital: a qualitative study*	Uganda	Low	Qualitative, phenomenological study using in-depth interviews	To document the lived experiences of pregnancy among women with sickle cell disease through in-depth, detailed narratives.	15 women with SCD including 12 with experience of previous pregnancy and 3 who were pregnant at the time of interview	B
Ulph F, Cullinan T, Qureshi N. et al. 2021 [37]. *Familial influences on antenatal and newborn haemoglobinopathy screening*	UK	High	Cross-sectional, qualitative study using semi-structured interviews	To examine service users’ prior knowledge, screening decisions and adaptation following HB screening within the context of social and familial influences	37 women and men including couples (28 Women) receiving carrier results for themselves or their child after universal haemoglobinopathy screening (including newborn). Family origin described as African/African Caribbean (14; Indian Sub-continent (10); Middle East (4); SE Asia & China (2); White European (6); Mixed (1)	B+
Xavier ASG, Ferreira SL, Carvalho ESS et al. 2013 [38]. *Perception of women suffering from sickle cell anemia regarding pregnancy: an exploratory study*	Brazil	Upper-Middle	Qualitative and descriptive using individual semi-structured interviews	To analyze the perception of women suffering from sickle cell anaemia regarding pregnancy	25 women with sickle cell anaemia reflecting on pregnancy and childbirth. 56% stated they were black and 44% mixed skin colour. 56% had been pregnant more than once and 72% had experienced complications during pregnancy	C+

### Findings

Fourteen descriptive themes (review findings) were generated, most of which were graded as moderate or low using GRADE-CERQual. From these four analytical themes were developed: *Beyond the routine of antenatal screening; Myths, misunderstandings and submissiveness; Fear and uncertainty in the face of adversity; The importance of familial and organizational support.* The descriptive themes, analytical themes and associated GRADE-CERQual assessments are shown in Table 3 below.

**Table 3:** Descriptive and analytical themes.

Descriptive theme	Contributing Studies (Ref’s)	CERQual Grading	Supporting Quote(s) and Ref	Analytical Theme
**Importance of screening women early:** Both women and health professionals supported early screening for SCD or SCT with some health professionals endorsing universal screening at an early stage. Some women advocated screening prior to the development of relationships to ensure SCD compatibility, while others favoured preconception screening or early pregnancy screening to give them time to make decisions (if required).	4 Studies – 29, 31, 34, 35.	Low	*“I think we should have screening the ﬁrst time we see the doctor. I was screened at the end of my third or fourth month of pregnancy and the results said I was a carrier. They called my husband and by then I was almost 4 months pregnant. I was worried that if my husband had the trait I might have to go for an abortion… at four, 5 months[gestation] its very hard for anyone* [35] *(P12)”If I had known right from the start that I was a carrier, I would have taken more care over my partner. I think I would have chosen a different partner.”* [31]	**Beyond the routine of antenatal screening**
**Screening as a moral imperative:** Women often couched their responses to screening questions in terms of ‘being a good mother’. For some women the desire to have a healthy baby influenced their decision to opt for prenatal screening, while for others the risks associated with foetal screening and the potential for harm (miscarriage), also based on the desire to have a healthy baby, prevented them from utilizing this test.	4 Studies – 28, 34, 35, 37.	Low	*‘Women want an explanation about the health of their baby, to know a little bit more about the health of the baby. I think that’s a priority when they come.’ [34] (HCP017) “I have talked to my partner, my family and friends and I just don’t want to have [a] test of [our] baby before birth [prenatal diagnosis]; there is a risk of miscarriage I just said no I don’t want to take that risk...* P12 [37]	
**Coping with the unexpected consequences of routine antenatal screening:** Some women felt unprepared for a positive antenatal screening result either because the test was presented to them as ‘routine’ or because they did not consider themselves to be at risk. This sometimes left them feeling shocked, angry and upset and they recounted difficulties in making decisions they had not expected to make. Health professionals also described difficulties in dealing with women’s emotional responses and the challenges of explaining potential implications.	7 Studies – 28, 29, 30, 31, 34, 35, 37	Moderate	*“We’ve had mixed reactions. Usually the reaction is, ‘well this is usually a black blood problem, so how come you’re telling me, I’m white, that I’m a carrier for this thing’? And usually shock. And in some cases anger and disbelief”. Health Professional,* [29]*. “We’ve had your blood results back and I can tell you that you’ve got sickle cell, I’m a sickle cell carrier. And at that stage I was really shocked ’cos I thought, I still didn’t think I’d have it ’cos my sister hasn’t got it or anybody so...”* P9 [37]	
**Doubts about ability to become pregnant based on myth and misinformation:** Beliefs about pregnancy were often shaped by socio-cultural attitudes and the (mis)understanding that women with SCD were unable to conceive or, more explicitly, should not conceive. Women who accepted these beliefs reported doubts about their ability to become pregnant and, as a result, did not use contraception. This sometimes led to unintended and/or unwanted pregnancies.	3 Studies – 28, 32, 36.	Very Low	*“I didn’t avoid having a child. The gynecologist said I wasn’t very fertile, so I thought I couldn’t have a child. The gynaecologist said that the patient with sickle cell anaemia couldn’t get pregnant and, sometimes, need to undergo a treatment”.* [32] *“I’ve never used contraceptives, it’s been a long time since I’ve had sex, but I’ve never been pregnant. I didn’t expect to get pregnant... One day, I got pregnant.”* [28]	**Myths, misunderstandings and submissiveness**
**Lack of knowledge and information about SCD and screening:** Women and health professionals reported a lack of knowledge about SCD and highlighted the need for more information and professional training. Specifically, women wanted more information about the need for screening, the meaning of ‘carrier status’ and SCD in general presented in an easy-to-understand format with diagrams and infographics. Staff, outside of specialist centres, required more training on knowledge of SCD as well as training in cultural competence.	7 Studies – 29, 30, 31, 34, 35, 36, 37.	Moderate	*“I just found out that I was a carrier so I really want to know more about it, whether it’s going to affect my baby or not, that’s what I’m really concerned about. The doctor didn’t give me that information* (P8) [35]. *“Midwives have a set patter, try to get it over with immediately, not confident to answer questions on sickle cell/thalassaemia”.* Researcher notes; [30] *“Of course, the medical people we meet many times they don’t understand sickle cell and yet they are the medical people who should know the condition but they don’t understand*.” [36]	
**Deferential attitude towards health professionals leads to directive care:** A tendency to defer to the views of health professionals, especially doctors, meant that women were less likely to engage in informed or shared decision making when making screening decisions or choices about their care. In some cases, this view was perpetuated by health professionals. Some women also felt that screening choices or counselling services were directive, and, in one instance, a termination was carried out without the woman’s consent.	4 Studies – 31, 34, 35, 36.	Low	*“Generally, patients go on what we say anyway because in a lot of cultures, the doctor still knows best.”* (HCP020). [34]. *“I don’t think the doctor gave a choice. The doctor said go tomorrow for this test. I’m happy because it’s beneficial for us. That’s why he’s saying that” (P12)*. [35]	
**Fatalistic beliefs lead to passivity and conflict:** A belief in the will of God to determine outcomes sometimes led to passivity in women’s decision making with some willing to accept having a child with SCD as part of ‘God’s plan’. For others, the decision to decline foetal testing was based on religious opposition to the procedure and, in some instances, religious views on pregnancy termination caused conflict and turmoil when this possibility arose.	4 Studies – 31, 34, 36, 38	Low	*“There wasn’t any point in getting checked, because neither did we want to have a termination and neither did we think there was any point … I left it to Allah... The individual cannot do anything”.* [31]. *“..... I said: ‘Oh my God, forgive me, I know it is a sin, but help me losing this child.’ Then I said I was not going to have this baby. God forgive me, because I know it’s a sin, but I don’t want it. I have already suffered a lot. I thought about it, but I was afraid. I felt guilty”*. DSC3 [38]	
**Language barriers hinder communication:** In situations where women’s first language wasn’t the local norm, they recounted difficulties in understanding SCD, why they were being screened and the implications of the screening results. Health professionals recognized these challenges and also highlighted the lack of translators and/or interpretation services in some contexts.	4 Studies – 29, 31, 34, 35.	Low	*“I would like to know more about whether it is a serious matter because I don’t understand very well English. I didn’t really know what was going on. It was a surprise when they told me I had the condition because I never heard about it and I would have liked to know more about it. They can’t explain to me everything, I asked them to send me an interpreter but they couldn’t help me”* (P1). [35]	
**Stigmatization, discrimination and insensitivity:** Pregnant women with SCD or SCT sometimes experienced stigmatization from health professionals, family members and the wider community largely because of a belief that they should not conceive. Some women chose to keep their pregnancy secret or only inform trustworthy, supportive family members to avoid judgement. In addition, health professionals screening practices often seemed to be based on preconceived and poorly informed notions of SCD prevalence, race and ethnicity with some finding it difficult to query women’s ethnicity or race without appearing ‘racist’.	7 Studies – 29, 30, 33, 34, 36, 37, 38.	Moderate	*“When I went to the hospital for the first time, the doctor that attended to me told me not to get pregnant again and she went on to tell me I shouldn’t have gotten pregnant because sicklers don’t give birth and die at the time of delivery.”* P12 [36]. *“I cried because I didn’t want anyone to know. I mean I wanted everyone to rejoice with me that I have a baby and I didn’t want anyone to say ‘Oh that baby could be a carrier’”*. [37] “*The midwives that I spoke to, they had difficulty in sometimes asking the woman their ethnicity or if they were given an answer that they weren’t sure fitted with what they felt the individual was, they had difficulty probing that to get more detailed information. Sometimes the midwives actually made assumptions”.* Nurse Counsellor [29].	**Fear and uncertainty in the face of adversity**
**Pregnancy is marked by painful and uncomfortable eruptions of SCD symptoms:** Women with SCD experienced acute flare-ups of SCD symptoms during pregnancy including severe pain, infection, fever and sometimes admission to hospital for blood transfusions. Most women tolerated these symptoms because of the overwhelming desire to be a mother and some regarded the severity of SCD pain as preparation for the pain they expected to experience during childbirth.	3 Studies – 28, 36, 38.	Very Low	*“I realized I was pregnant when I had more bouts of pain. I’ve had three seizures during this pregnancy.”* [28]. “*My pregnancy was complicated. I felt so much pain, too much pain indeed. I took medication during the whole pregnancy [...]. I had much trouble, I was hospitalized; was in great pain; I had a urinary tract infection, fever and pain*.” (CSD 1) [38]	
**Intense feelings of fear, guilt and anxiety throughout the maternity journey:** For women with SCD the maternity journey was accompanied by intense feelings of fear and anxiety. These related to the current and future wellbeing of the baby, whether he/she would suffer with SCD and whether they would be able to care for their baby after birth. Women also worried about pregnancy related increases in SCD symptoms and struggled with thoughts of dying during labour or childbirth. Being regarded as ‘high risk’ amplified these feelings. Some women also experienced guilt or regret about becoming pregnant, their choice of partner and passing on SCD to their baby.	5 Studies – 28, 31, 32, 36, 38	Moderate	*“I am very anxious all the time. I’m worried about whether my baby will be disabled......I’m so scared that I don’t tell anyone. I feel trapped. I don’t want to have a baby who isn’t healthy.... I pray a lot that the baby doesn’t die or be born disabled like I am.”* [28]. *“You have to handle everything. Everything depends on you. I started getting scared of having a sick child; so much that, when I see a scene of a child with any problem, I don’t look at it”.* [38]. *“I was just afraid that he was born with my same problem, that’s my feeling, fear of giving birth to a child equal or worse than me”. [32]*	
**The joy of achievement and despair of loss:** Women spoke about their journey to motherhood in terms of a struggle through physical and emotional pain culminating in feelings of triumph after the successful birth of a healthy baby. Some women also felt that pregnancy normalised their situation (being seen as a pregnant woman rather than a woman with SCD) while others felt joy and relief when their baby was born without SCD. In contrast, women who experienced pregnancy loss or had to terminate their pregnancy felt deep despair, trauma, grief and extended periods of depression.	4 Studies – 32, 33, 36, 38.	Low	“*A good feeling, because I’ve been through all this [Pregnancy & childbirth]. My daughter is fine and I will grow her. But now the feeling refers to relief, happiness. Of going through it all and being fine. A feeling of victory”*. (I 11) [32]. *“It is a huge sadness to lose a child, I went into depression for months I was locked in a room. At first, guilt came, because I didn’t think it was an abortion and I kept putting on strength”* (DCS2) [34]	
**Influence of family, professional and social support:** Women valued the support of partners, family members and health professionals during their maternity journey. When support was not forthcoming women experienced feelings of loneliness and isolation and occasionally considered termination. Some women were also wary of revealing their +ve carrier status before or during pregnancy in case it affected their relationship with their partner or family support networks.	5 Studies – 32, 33, 36, 37, 38.	Moderate	*“It was difficult for me. My family didn’t support me. The child’s father didn’t care, I went to the hospital alone, often, and I sat there alone, without any help, so it wasn’t a very good experience, indeed”*. (I 08) [32]. *“They tell us that we can’t but me I have seen that if you have a good support, and when you are getting support, you are cared for, then you can carry the pregnancy up to term, it is only care and love. When I was weak, he [partner] was there for me.”* P4. [36]	**The importance of familial and organizational support**
**Infrastructure and organizational issues impact on quality of care:** Both women and health professionals felt they needed more time to discuss SCD and screening for SCD during the antenatal period. Women preferred to receive a + ve screening result in person to check understanding, discuss implications and make plans and decisions. Health professionals preferred to work in small teams to aid continuity and build relationships with women. Some women also highlighted the importance of specialist SCD centres but bemoaned the lack of beds in these facilities. In some contexts, delays in receiving care meant extended and unnecessary periods of pain though delays could be circumvented through bribes and informal payments (for those with sufficient resources)	7 Studies – 29, 30, 31, 34, 35, 36, 38.	Low	“*I think also they’re going to need time [Health professionals], they will have to be given extra time to do the screening, so that they can explain fully to the client why they’ve been screened.”* SCD Nurse Counsellor [29]. *“So when I went to the hospital, there is also our clinic, a sickle cell clinic. There is a doctor. He connected me to a specialist that I went to see. When I went, he told me some things and I did them”* P2. [36]	

### Beyond the Routine of Antenatal Screening

This analytical theme incorporates three of the descriptive themes relating to ‘the importance of screening women early’; ‘screening as a moral imperative’ and ‘coping with the unexpected consequences of routine antenatal screening’.

The importance of screening for SCD before reproductive decision making was highlighted by a number of women in HICs [29,31,34,35]. Some women wanted to be screened for SCD status before the development of relationships to avoid awkward conversations with prospective partners [31,35]. This was particularly important for women with SCT who felt that earlier screening (before antenatal care) might have prevented them from engaging in relationships with partners who were also carriers [31,35]. For others already in relationships, there was a belief that pre-pregnancy screening would have given them (and their partners) time to make important decisions about reproductive healthcare [35]. In HIC contexts some health professionals felt that universal screening, rather than targeted screening for haemoglobinopathies (including SCD and Thalassemia), should be available for all women during the early antenatal phase [29].

Women sometimes couched their responses to screening in terms of ‘being a good mother’ [28,34,35,37]. For some this meant accepting all screening opportunities, including foetal screening, in the understanding that they were making the safest choices to try and ensure the best possible outcomes for themselves and their baby [34,35]. For others, the safest choice sometimes meant declining a screening test, particularly a foetal screening test, because of the potential risks to their baby [37]. In both situations the women were expressing their perception of what a good mother should be, but they arrived at different conclusions as a result.

Some women also felt unprepared for antenatal SCD screening tests and expressed shock and surprise when they received a positive result [28–31,34,35,37]. Usually, this was because they did not consider themselves to be at risk of SCD [28–31,34,37]. However, some women did not really understand what they were being tested for or the test had been presented to them as ‘routine’ and therefore nothing to worry about [30,31,35]. A positive antenatal screening test was particularly challenging for women identifying as ‘white’ because they were under the impression that SCD was not a disease they could inherit [29,37]. Health professionals also reported difficulties in explaining a positive result to women who, for whatever reason, didn’t expect to receive one [29,34]. This was particularly apparent when health professionals had to discuss the implications of a positive result and the various choices available, including the possibility of terminating a pregnancy [34].

### Myths, Misunderstandings and Submissiveness

This analytical theme is made up of five descriptive themes, namely, ‘doubts about ability to become pregnant based on myth and misinformation’; ‘lack of knowledge and information about SCD and screening’; ‘fatalistic beliefs lead to passivity and conflict’; ‘deferential attitudes towards health professionals leads to directive care’ and ‘language barriers hinder communication’.

For some women with SCD the very notion of becoming pregnant was obscured in a cloud of myth and misinformation [28–32,34–38]. In some contexts, socio-cultural understandings of SCD fostered the belief that women with the condition could not become pregnant, leading to the assumption that contraception was not important [28,32,36]. Such beliefs occasionally resulted in shock and surprise when an unintended and/or unwanted pregnancy came to light [28,32]. In other settings the prevailing socio-cultural belief was that women with SCD should not become pregnant, largely based on the view that it would be amoral or cruel to pass on the condition to a child [36]. This latter view was sometimes supported by health professionals who expressed their concerns about the health and welfare of mother and baby by blaming and shaming the women in their care for becoming pregnant [36].

The myths associated with SCD and conception appeared to be born out of a lack of knowledge and information [29–31,34–37]. Women in a variety of settings felt they did not know enough about SCD and lacked understanding about basic genetics, screening practices and the implications of being a carrier [31,35,37]. These issues were compounded for those who were not fluent in the local native language, especially where translation or interpretation services were inadequate or unavailable [29,31,34,35]. Some women with SCD also found that health professionals lacked knowledge about the condition and were not able to offer advice and guidance about how to manage their pregnancies [29,35,36]. This was confirmed by health professionals in several settings who felt they needed more training to better understand the condition and how it affected women during pregnancy [29,30,36]. Other health professionals involved in antenatal screening in high-income settings (HICs) also felt awkward and uncomfortable asking questions about race and ethnicity during SCD screening and wanted more training in cultural awareness [29,30]. Arguably, because of a lack of knowledge about their condition or their carrier status, women in several contexts exhibited a deferential attitude to the views and opinions of health professionals [31,34–36]. In some instances this view was maintained or encouraged by health professionals leading to directive care and poor engagement in shared decision-making [34–36]. Some women also adopted a passive or fatalistic attitude to decision-making by deferring their choices around screening or potential termination to ‘the will of God’ [31,34,36,38]. Views influenced by religious beliefs also had an impact on acceptance or opposition to interventions like foetal diagnostic testing and created mental and emotional turmoil in some women who decided to terminate a pregnancy [38].

### Fear and Uncertainty in the Face of Adversity

This theme includes four descriptive themes, namely, ‘stigmatization, discrimination and insensitivity’, ‘pregnancy is marked by uncomfortable and painful eruption of SCD symptoms’; ‘Intense feelings of fear, guilt and anxiety throughout the maternity journey’ and ‘the joy of achievement and despair of loss’.

Women with SCD recounted an exhausting array of physical, practical, emotional and psychological challenges that impacted on their experiences of maternity care [28–34,36–38]. In navigating these challenges women displayed remarkable resilience and determination in order to fulfil their dream of becoming a mother. For some, the confirmation of pregnancy generated powerful emotions, eliciting feelings of guilt, fear and anxiety [28,32,33,38]. Women described feeling particularly anxious about the wellbeing of their baby and, where the foetal SCD status was unknown, whether the baby would be born with the condition [28,33,38]. In some instances these concerns persisted throughout pregnancy and prompted more apprehension about their ability to cope and, in some extreme examples, the prospect of dying during childbirth [32,38]. Women also worried about their future and the potential burden of having a child with SCD as well as their long-term ability to look after a child as a mother with a serious health condition [28,32]. In some contexts, women with SCD felt compelled to keep their pregnancy secret to avoid feeling stigmatized or judged by family members, the wider community and, occasionally, health professionals [36–38]. Health professionals, in turn, struggled with their own preconceptions and poorly informed notions of SCD prevalence and ethnicity and, in screening situations, made assumptions (sometimes incorrectly) about susceptibility to SCD based on appearance [29,30,37]. In some HIC settings these practices were perceived as discriminatory by the women being screened [33,37]. Pregnancy also prompted women to reflect on their reproductive choices and some expressed guilt about becoming pregnant and/or experienced regret about their choice of partner [31,32].

In addition to these emotional struggles, pregnancy often precipitated a flare-up of SCD related symptoms and women described experiencing acute pain, fevers and infections [28,36,38]. Sometimes these crises required hospital treatment and occasionally admission to hospital for blood transfusions [28,36,38]. Despite these practical, emotional and physical challenges women with SCD expressed an overwhelming desire to become a mother [31,32]. A few felt that pregnancy ‘normalized’ their condition, enabling them to be perceived as ‘normal women’, capable of carrying a pregnancy, rather than ‘sick women’ who couldn’t or shouldn’t conceive [38]. For some, the successful birth of a healthy child elicited feelings of incredible relief and, ultimately, a sense of triumph or victory [32,38]. For others, the profound sense of loss following a miscarriage or a pregnancy termination, generated feelings of despair, trauma and grief sometimes leading to prolonged periods of emotional turmoil or depression [33,36,38].

### The Importance of Familial and Organizational Support

This analytical theme is comprised of two descriptive themes, ‘influence of family, professional and social support’ and ‘infrastructure and organizational issues impact on quality of care’.

In various contexts women with SCD recalled the importance of support during their maternity journeys [29–38]. In most cases this related to support from families and, in particular, the support of their partner [32,33,36–38]. Despite the challenges of carrying a pregnancy women remarked on the strength they received from having a supportive partner and highlighted the importance of feeling loved and cared for in these circumstances [33,36,37]. In other contexts, however, partners were less supportive and women recalled feeling rejected or abandoned by a partner when the foetus was confirmed to have SCD [32,37,38]. In addition, some pregnant women (with the condition) felt isolated by their families and/or communities because of the associated stigma [37,38]. This sometimes generated feelings of loneliness and depression and, in these circumstances, women began to doubt their ability to cope and considered pregnancy termination [38]. In other contexts, women kept their carrier status secret in order to avoid feelings of rejection or stigmatization and to maintain family support networks [37,38].

Pregnant women with SCD also valued the support of health professionals and highlighted the importance of empathy, understanding and respect during their maternity journey [36]. Women also appreciated the support, advice and expertise of health professionals working in specialist SCD clinics or in hospitals with SCD support services (where available) [36]. However, some women highlighted the lack of beds in specialist centres and delays in receiving support and care leading to extended periods of pain and discomfort while waiting to receive treatment [38]. In one setting access to specialist services appeared to be facilitated by informal payments resulting in an inequitable service where women without the required finance were unable to receive support and treatment [36]. Women also remarked on the lack of time spent with health professionals during consultations or antenatal appointments [35]. Women wanted to receive more information and advice about SCD screening or the opportunity to discuss their pregnancy or birthing options in more detail [31,35,37]. Health professionals also highlighted the limited amount of time they were able to spend with women during screening sessions or at antenatal appointments and the difficulties of discussing quite complex issues within a 15–20 minute time slot [29,30,34].

## Discussion

Findings indicate that for women with SCD the journey to motherhood can be an arduous process involving a series of emotional, physical and practical challenges above and beyond those experienced by women in less vulnerable circumstances. In addition to the pre-existing challenges associated with having a chronic health condition, women with SCD may encounter misinformation, stigmatization, discrimination and judgement as well as acute flare-ups of debilitating SCD symptoms. Pregnant women with SCD may also experience heightened levels of anxiety because of concerns about their own vulnerability, the status of the foetus and their future capacity to look after a baby. Despite these challenges the review suggests that pregnancy can ‘normalize’ women’s feelings about their condition leading to a belief that they can carry a pregnancy and give birth to a healthy child like other (healthy) women without SCD.

Given the increasing numbers of women with SCD reaching reproductive age [8] it is vital that education initiatives are prioritized to improve understanding, address stigmatization and support women in their reproductive choices. This is particularly important in regions of Sub-Saharan Africa where the disease burden is highest and resources may be limited [39]. In these settings where engagement with health providers, particularly during antenatal care, may be low or infrequent, a community focus is likely to be more successful [40,41].

For women diagnosed with SCT following antenatal screening appointments findings illustrate the sense of shock and surprise when an unexpected result is confirmed, especially when the test is presented to them as ‘routine’. Poor understanding of the test and its implications as well as a general lack of knowledge about the genetic origins of the disease highlight notable deficiencies in women’s understanding of SCT and the need for more relevant, understandable, information resources. In addition, findings from this review suggest that women diagnosed with SCT during antenatal screening would prefer to take the test prior to conception so that reproductive planning and decision-making can occur before they become pregnant. Pre-relationship or pre-marital screening for SCD has been introduced successfully in some European countries (e.g., Greece and Cyprus) though it may not be suitable in all contexts where the identification of carrier status can lead to discrimination or stigmatization [42]. Although all of the included studies exploring haemoglobinopathy screening were conducted in a UK context, (where a specific haemoglobinopathy screening programme has been in place for almost twenty years [43]), the findings may translate to other HIC settings with similar health systems and populations.

As noted above, and in accord with evidence from survey-based studies in both LMICs [14,44] and HICs [16,45], findings in this review highlight a lack of knowledge about SCD and SCT amongst populations affected by the disease. Poor understanding of the condition in the general population may contribute to the stigmatization of individuals with SCD [21,46] and, as the findings illustrate, lead to discrimination against women who wish to conceive.

The review findings also indicate a need for more education amongst health professionals especially in areas where the disease is prevalent or in settings where there are populations likely to be affected by the disease. Recent reviews of studies exploring the impact of SCD focused educational interventions for healthcare professionals found that provider understanding of the condition could be improved using a variety of educational initiatives [47,48]. Importantly, the authors also identified studies which highlighted improvements in provider attitudes towards people with SCD [47,48]. Although this is encouraging, neither review identified any studies conducted in a maternity context where specific knowledge gaps around screening for SCT and the impact of pregnancy on women with SCD (and vice-versa) remain. This highlights the need for the development of bespoke SCD focused training courses for health professionals working with women (and their families) in reproductive and maternal health.

In parts of the world where SCD is endemic, particularly in Sub-Saharan Africa, much of the focus has concentrated on newborn screening and the creation of national registers to map local prevalence and monitor infant health [49]. Pilot newborn screening programmes have been introduced in a number of African countries with high levels of SCD including Ghana, Uganda, Tanzania, Senegal and Nigeria [50]. The success of such programmes in LMICs largely depends on the willingness of governments to prioritize SCD amidst the myriad of competing healthcare priorities, as well as a commitment to invest in resources and infrastructure to support the programme. A recent study exploring some of the family, community and health system considerations for newborn haemoglobinopathy screening programmes in Uganda suggests investment is required to support public awareness campaigns to reduce stigma and provide information about access to services and potential treatments [51]. The same study also highlights further investment is needed in testing facilities (and associated infrastructure), the training of health professionals and the establishment of specialist SCD centres in areas of high prevalence [51].

In addition to these health system requirements this review also identified a variety of socio-cultural and ethical considerations associated with antenatal haemoglobinopathy screening. The tendency for some women to defer to the views of health professionals when making screening decisions raises questions about individual autonomy and informed consent. While some women may be willing to accept and act on the views of a health professional in light of their medical expertise, this passive approach can lead to a pattern of directive care in which the views of the health professional hold sway. As illustrated in a systematic review exploring some of the cultural and ethical implications of clinical genetic testing in LMICs [52] this may be of particular concern if, for example, a health professional holds a specific view on selective termination of pregnancy following a positive foetal screening test. Similarly, this review also found that some health professionals withheld or minimized important information about haemoglobinopathy screening, possibly to save time or avoid complicated discussions about genetics or ethnicity. As a result women lacked advocacy and, as noted above, reported feeling shock and anger when the results of an antenatal screening test came back positive. This issue touches on some of the sensitivities of screening for a genetic condition that is historically associated with specific population groups whereby SCD “*becomes socio-politically allied to ideas of repair, in terms of the state improving the health of a neglected ethnic minority population”* [53]. There is a moral argument that screening for a potentially life-threatening condition in often underserved communities is an ethically responsible thing to do. However, findings from this review indicate that doing so with arbitrary (and sometimes discriminatory) ideas of who should be screened and not providing appropriate time or information to those being screened is unjust and unethical.

### Limitations of the Review

Although the impact of SCD during pregnancy and childbirth is more common in Sub-Saharan Africa, the Middle East and parts of South-East Asia this review only found one relevant study, conducted in Uganda [35]. This is a limitation as there are likely to be additional and/or more nuanced socio-cultural and socio-economic considerations in other contexts, particularly in LICs where resource constraints and competing healthcare priorities may impact on SCD service delivery and engagement.

Given that the other ten studies in the review were conducted in HICs or Upper-Middle Income countries where SCD services are relatively well established, findings largely reflect the views of a particular population of women with access to SCD screening programmes, health surveillance systems, treatments and specialist services that may not be available in resource-limited settings. In addition, nine of the eleven included studies were conducted in just two countries, six in the UK and three in Brazil, and only one [33] was conducted within the last five years. This rather jeopardizes the transferability and generalizability of the findings and also highlights the paucity of research in this area. Further research exploring the experiences of pregnant women with SCD, especially in high burden countries may provide valuable insights into how they can be better supported during their maternity journey.

The review also identified only three studies reporting on the views of healthcare providers (all from the UK), and all were focused on their experiences of providing antenatal screening for SCD. This is another limitation of the review and further undermines the transferability and generalizability of the findings to other contexts. Health system structures, approaches to screening and treatment options for pregnant women are likely to be different in other countries, particularly in LMICs, where a lack of resources, infrastructure and understanding of the condition may impede the provision of quality maternity care for women with SCD or SCT.

Given the paucity of studies in this area and the limitations outlined above it is important that further qualitative research is conducted in areas where SCD is prevalent and where understanding of the condition is poor. More up to date studies focused on women’s experiences of SCD or SCT during their engagement with maternity services are likely to highlight current needs and concerns for this population of women. In addition, qualitative studies with maternity providers working in areas where SCD is prevalent may be able to offer further insights into their educational needs, resource requirements and attitudes towards SCD as well as their perspective on supporting a group of women with specific care requirements.

## Conclusion

For women with SCD the journey to motherhood can be a formidable endeavour, beset with pain, anxiety, uncertainty and sometimes tragedy. Given the increasing numbers of women with SCD reaching reproductive age it is vital that healthcare services are better equipped to meet their specific needs. Educational initiatives aimed at health professionals may help to increase knowledge of SCD and its impact on pregnancy, while community focused public awareness campaigns may generate more understanding of the condition, dispel myths and reduce stigma.

To further reduce the impact of SCD in areas of high prevalence, SCT screening programmes offer opportunities for women (and their families) to play an active role in reproductive decision making. Pre-marital or pre-conceptual screening for SCT may be appropriate in some contexts but must be considered alongside the potential for stigmatization. In addition, it is important that screening services are properly resourced and able to provide understandable information in a supportive, non-judgemental environment with well-trained staff sensitive to the needs of women from a variety of different ethnic and cultural backgrounds.

## Supporting information

S1 Appendix Search startegies for each database. (XLSX)

S2 Appendix Data extraction, analysis, synthesis and CERQual grading for included studies. (XLSX)

S3 Appendix List of articles retrieved from our searches. (XLSX)

S4 Appendix List of included and excluded studies. (XLSX)

S5 Appendix PRISMA Checklist. (DOCX)

## Abbreviations

CINAHL: Cumulative Index to Nursing and Allied Health Literature
CNKI: China National Knowledge Infrastructure
EMBASE: Excerpta Medica dataBASE
GRADE-CERQual: Confidence in the Evidence from Reviews of Qualitative Research
HIC: High Income Country
LIC: Low Income Country
LMIC: Low or Middle Income Country
MEDLINE: Medical Literature Analysis and Retrieval System Online
MIDIRS: Maternity and Infant Care database
NCD: Non-Communicable Disease
PRISMA: Preferred Reporting Items for Systematic reviews and Meta-Analyses
SCD: Sickle Cell Disease
SCT: Sickle Cell Trait
WHO: World Health Organization
