# Supplementary material for: Sickle cell disease and maternity care: A qualitative synthesis of women’s and health providers experiences
Source: PLoS One. 2026 Jul 13;21(7):e0352992. doi: 10.1371/journal.pone.0352992 (PMC13362093; doi:10.1371/journal.pone.0352992)
Supplement: Attachment — Submitted filename: Response to Reviewers.docx [file pone.0352992.s007.docx]

**PONE-D-25-01416**

**Sickle cell disease and maternity care: A qualitative synthesis of women’s and health providers experiences.**

**Response to Reviewers**

Many thanks for taking the time to give our article your careful consideration. Detailed responses to all queries and concerns raised by the editor and reviewers are outlined below (in red font).

When submitting your revision, we need you to address these additional requirements.

1. Please ensure that your manuscript meets PLOS ONE's style requirements, including those for file naming. The PLOS ONE style templates can be found at 
<https://journals.plos.org/plosone/s/file?id=wjVg/PLOSOne_formatting_sample_main_body.pdf> and 
<https://journals.plos.org/plosone/s/file?id=ba62/PLOSOne_formatting_sample_title_authors_affiliations.pdf>

We have checked this guidance and believe our manuscript meets with journal requirements but are happy to accept further advice if this is not the case

2. Please remove all personal information, ensure that the data shared are in accordance with participant consent, and re-upload a fully anonymized data set.

We believe all data sets are fully anonymized. This is a review of existing, published, qualitative articles and not an empirical study so, as far as we are aware, participant consent is not required

Note: spreadsheet columns with personal information must be removed and not hidden as all hidden columns will appear in the published file.

We believe all spreadsheet columns are ‘clean’ and do not contain any personal information

3. As required by our policy on Data Availability, please ensure your manuscript or supplementary information includes the following:

A numbered table of all studies identified in the literature search, including those that were excluded from the analyses.

We have added an additional supplementary table (labelled S2 Appendix) listing all of the 47 studies identified in our searches (at full text stage) including the author(s), title, date of publication, country (and income level), population under investigation, whether the study was excluded or included and, where relevant, the reason for exclusion.

For every excluded study, the table should list the reason(s) for exclusion.

Reasons for exclusion have been added in the S2 Appendix, as noted above.

If any of the included studies are unpublished, include a link (URL) to the primary source or detailed information about how the content can be accessed.

All of the included studies are published and the references provided in the reference section. Findings from one study (Ref no 28) were extracted from a book and a hyperlink to the book source has been added to this reference. [Ethnicity and screening for sickle cell/thalassaemia : lessons for practice from the voices of experience / Simon M. Dyson. | Wellcome Collection](https://wellcomecollection.org/works/ty8pndkg)

A table of all data extracted from the primary research sources for the systematic review and/or meta-analysis. The table must include the following information for each study:

All of the information relating to the studies included in this review is located in the Excel spreadsheet labelled as ‘S3 Appendix’. This includes details of the study characteristics (page 1); a detailed quality appraisal of each study (page 2); the coding and data extraction of findings from each study (page 3); the final themes (page 4) and a GRADE CERQual appraisal (including a level of confidence grading) of the final themes on page 5. We believe this file makes our approaches towards data extraction, analysis and synthesis robust and transparent.

Name of data extractors and date of data extraction

We have added the names of the data extractors and the dates when data was extracted to the ‘Data Extraction and Analysis’ section of the manuscript on page 11.

Confirmation that the study was eligible to be included in the review.

This information is available in the supplementary files, S2 and S3 Appendix

All data extracted from each study for the reported systematic review and/or meta-analysis that would be needed to replicate your analyses. In a qualitative

From a qualitative perspective this information is reported clearly and transparently on pages 3 and 4 of the S3 Appendix

If data or supporting information were obtained from another source (e.g. correspondence with the author of the original research article), please provide the source of data and dates on which the data/information were obtained by your research group.

We didn’t retrieve any data from additional sources

If applicable for your analysis, a table showing the completed risk of bias and quality/certainty assessments for each study or outcome.  Please ensure this is provided for each domain or parameter assessed. For example, if you used the Cochrane risk-of-bias tool for randomized trials, provide answers to each of the signalling questions for each study. If you used GRADE to assess certainty of evidence, provide judgements about each of the quality of evidence factor. This should be provided for each outcome.

Since this is a qualitative systematic review we are not reporting on pre-determined quantitative outcomes. We used the GRADE-CERQual tool (described on pages 8-9 in the manuscript) to assess confidence (high, moderate, low or very low) in the review findings in accord with Cochrane approaches to the reporting of qualitative data from these types of reviews. The GRADE-CERQual assessments for each review finding (theme) are highlighted in Table 1 and the details of how these grades were achieved is displayed on page 5 of the S3 Appendix.

An explanation of how missing data were handled.

As far as we are aware there is no missing data

This information can be included in the main text, supplementary information, or relevant data repository. Please note that providing these underlying data is a requirement for publication in this journal, and if these data are not provided your manuscript might be rejected.

See notes above – we believe we have provided all underlying data

4. We note that the grant information you provided in the ‘Funding Information’ and ‘Financial Disclosure’ sections do not match.

When you resubmit, please ensure that you provide the correct grant numbers for the awards you received for your study in the ‘Funding Information’ section.

We have provided a financial statement at the beginning of this letter:-

“This work was commissioned to the University of Central Lancashire by the UNDP/ UNFPA/UNICEF/WHO/World Bank Special Programme of Research, Development and Research Training in Human Reproduction (HRP), a cosponsored program executed by the World Health Organization (WHO)”

5. Please review your reference list to ensure that it is complete and correct. If you have cited papers that have been retracted, please include the rationale for doing so in the manuscript text, or remove these references and replace them with relevant current references.

As far as we are aware we have not cited any publications that have been retracted.

Any changes to the reference list should be mentioned in the rebuttal letter that accompanies your revised manuscript. If you need to cite a retracted article, indicate the article’s retracted status in the References list and also include a citation and full reference for the retraction notice.

Additional Editor Comments:

Dear Author,

Thank you for submitting your manuscript for evaluation. However, in order to strengthen and clarify your manuscript, the following revisions are recommended:

Introduction Section:
You should clearly emphasize the necessity of your study and explicitly address the research gap to make the introduction more focused.

We have removed some superfluous text from the introduction, added some text to highlight the research gap and made the objectives of the study more clear (see pages 4 & 5 in the introduction section)

Methods Section:
Inclusion and exclusion criteria should be described in more detail. This is crucial for ensuring the reproducibility of the study.

In addition to the text under ‘Criteria for Inclusion’ on page 6 we have added a box (Box 1) listing the inclusion and exclusion criteria (see pages 7 & 8)

Discussion Section:
The discussion should be concise, and redundant points should be removed.

We have removed and/or summarized sections of text in the discussion to make it more concise (see pages 23-27)

Most of the included studies originate from the United Kingdom and Brazil. This may limit the generalizability of the findings to other regions, particularly to low-income countries (LICs), where SCD is more prevalent. This limitation should be explicitly acknowledged, and the potential variations in findings across different contexts should be discussed.

We have added a sub-title ‘Limitations of the Review’ on page 27 where the issues of generalizability and transferability are explicitly acknowledged.

Only three studies focusing on healthcare providers are included, all of which are from the United Kingdom. This limits the understanding of healthcare providers' experiences in different healthcare systems. This limitation should be addressed, and future research directions should be proposed.

This issue is acknowledged and addressed under the new sub-heading, ‘Limitations of the Review’ on page 27

The discussion should conclude with recommendations for future studies.

We have added a paragraph at the end of the discussion section with recommendations for future studies

Abbreviations Table:
To enhance readability, please provide a table listing the abbreviations used in the manuscript. We have added a List of Abbreviations after the Abstract

Limitations Section:
A separate section should be added to discuss the limitations of the study in greater detail.

As noted above we have added a Limitations section on page 27

References:
Since most of the references are older than five years, it is recommended to update some of them with more recent literature.

As outlined in our ‘Criteria for Inclusion’ (page 8) our searches were conducted during the period 2000-2024 to ensure our studies captured the views of the current generation of women (and health professionals). 10 of our 11 included studies (Ref’s 27-37) were conducted prior to 2019 with the oldest conducted in 2005 so this is something we can’t change. However, we appreciate your comment and have added a sentence to the ‘Limitations’ section to reflect this concern. With regard to the supporting references we have been through them all and note there are a few older ones (pre 2010) in the methods section which again we can’t change because they cite methodological papers as ‘benchmark studies’ for a particular methodology or approach to analysis/synthesis, i.e. refs 22,23 & 25. Of the remaining references most are current or refer to a specific (and relevant) set of data which, as far as we are aware, has not been updated recently (e.g. ref’s 2 & 6). In a general sense there are a limited number of studies exploring SCD in a maternity context and, given the increasing numbers of women with SCD (or SCT) reaching reproductive age, there is a need to shine a light on this issue to raise awareness with a view to improving services for this population of women. This is one of the main reasons for conducting this review. We hope this addresses your concerns but we would be happy to take further guidance if required.

Additionally, we encourage you to review your manuscript for compliance with PLOS ONE formatting and writing guidelines. A general revision regarding language and clarity would also be beneficial. Please submit a revised version with the implemented changes clearly indicated.

We believe this review meets PLOS ONE formatting and writing guidelines and we hope we have addressed any concerns in the revised version

Reviewers' comments:

Reviewer's Responses to Questions

**Comments to the Author**

1. Is the manuscript technically sound, and do the data support the conclusions?

The manuscript must describe a technically sound piece of scientific research with data that supports the conclusions. Experiments must have been conducted rigorously, with appropriate controls, replication, and sample sizes. The conclusions must be drawn appropriately based on the data presented.

Reviewer #1: Yes

Reviewer #2: Yes

2. Has the statistical analysis been performed appropriately and rigorously?

Reviewer #1: Yes

Reviewer #2: N/A

3. Have the authors made all data underlying the findings in their manuscript fully available?

The [PLOS Data policy](https://eur01.safelinks.protection.outlook.com/?url=http%3A%2F%2Ftrack.editorialmanager.com%2FCL0%2Fhttp%3A%252F%252Fwww.plosone.org%252Fstatic%252Fpolicies.action%2523sharing%2F1%2F010f019511d601f4-8eb9054c-bf2a-4bf2-9eba-6aef8c11a098-000000%2F-f5q3qkXTtsQO6_CTcDcysd5ebLzGqNWE13QFD6YGVI%3D198&data=05%7C02%7Ckwfinlayson1%40uclan.ac.uk%7C30083b1d219044fcb2de08dd4efeb052%7Cebf69982036b4cc4b2027aeb194c5065%7C0%7C0%7C638753578260869757%7CUnknown%7CTWFpbGZsb3d8eyJFbXB0eU1hcGkiOnRydWUsIlYiOiIwLjAuMDAwMCIsIlAiOiJXaW4zMiIsIkFOIjoiTWFpbCIsIldUIjoyfQ%3D%3D%7C0%7C%7C%7C&sdata=HimmO7Fof5bIIvH22ifN53G%2Fh8ztBgPSUw1K5JlXDkQ%3D&reserved=0) requires authors to make all data underlying the findings described in their manuscript fully available without restriction, with rare exception (please refer to the Data Availability Statement in the manuscript PDF file). The data should be provided as part of the manuscript or its supporting information, or deposited to a public repository. For example, in addition to summary statistics, the data points behind means, medians and variance measures should be available. If there are restrictions on publicly sharing data—e.g. participant privacy or use of data from a third party—those must be specified.

Reviewer #1: No

We believe we have supplied all supporting data – either in the manuscript or in the S1, S2 or S3 appendices

Reviewer #2: Yes

4. Is the manuscript presented in an intelligible fashion and written in standard English?

PLOS ONE does not copyedit accepted manuscripts, so the language in submitted articles must be clear, correct, and unambiguous. Any typographical or grammatical errors should be corrected at revision, so please note any specific errors here.

Reviewer #1: Yes

Reviewer #2: Yes

5. Review Comments to the Author

Please use the space provided to explain your answers to the questions above. You may also include additional comments for the author, including concerns about dual publication, research ethics, or publication ethics. (Please upload your review as an attachment if it exceeds 20,000 characters)

Reviewer #1: Dear Author
Thank you for submitting your article for review
Your study is very valuable. Thank you
The following comments are provided to improve the research:
In the introduction section, the necessity of the work should be clearly stated.

We have amended the Introduction section to highlight the evidence gap more clearly and have outlined the objectives of the study towards the end of the Introduction (see pages 5 & 6)

In the methods section, the inclusion and exclusion criteria of the study should be more precisely stated.

We have added a box (Box 1) where the inclusion and exclusion criteria are clearly stated (see pages 8 & 9)

The discussion should be strengthened.

We have amended the Discussion section by removing some superfluous text and clearly stating the limitations of the review under a separate heading.

At the end of the discussion, suggestions for future studies should be mentioned.

We have also added some text at the end of the discussion highlighting some areas for future research (page 29).
Thank you

Reviewer #2: Thank you for your qualitative paper on: Sickle Cell Disease and Maternity Care: A Qualitative Synthesis of Women’s and Health Providers’ Experiences
Please find my comments

1- Introduction could be focused, addressing the research gap as well.

We have amended the Introduction section to highlight the evidence gap more clearly and have outlined the objectives of the study towards the end of the Introduction (see pages 5 & 6)

2- Discussion needs to be concise, some points are repeated .

We have amended the Discussion, removed some superfluous text and tried to remove any repetitive narrative from this section. We hope it is now more concise.

3- The majority of the included studies are from the UK and Brazil, which limits the transferability of the findings to other regions, particularly low-income countries (LICs) where SCD is highly prevalent. The authors should acknowledge this limitation more explicitly and discuss how the findings might differ in other contexts.

We have added a separate heading, Limitations of the Review (page 27), and discussed how the findings may differ in other contexts, (especially LICs) within this section

4- The manuscript includes only three studies focusing on healthcare providers, all from the UK. This limits the understanding of provider experiences in different healthcare systems. The authors should discuss this limitation and suggest areas for future research.

This is now addressed under the ‘Limitations of the Review’ section and suggestions for future research are highlighted beneath this heading (at the end of the discussion)

5- Please provide a table for abbreviations.

We have added a List of Abbreviations under the Abstract on page 4

6- Please provide a section for limitations.

As noted above we have added a heading, Limitations of the Review’, on page 27.

7- Please update some of your references, as most of your references are more than 5 years outdated.

As outlined in our response to the editor - Our searches were conducted during the period 2000-2024 to ensure our studies captured the views of the current generation of women (and health professionals). 10 of our 11 included studies (Ref’s 27-37) were conducted prior to 2019 with the oldest conducted in 2005 so this is something we can’t change. However, we appreciate your comments and have added a sentence to the ‘Limitations’ section to reflect this concern. With regard to the supporting references we have been through them all and note there are a few older ones (pre 2010) in the methods section which again we can’t change because they cite methodological papers as ‘benchmark studies’ for a particular methodology or approach to analysis/synthesis, i.e. refs 22,23 & 25. Of the remaining references most are current or refer to a specific (and relevant) set of data which, as far as we are aware, has not been updated recently (e.g. ref’s 2 & 6). In a general sense there are a limited number of studies exploring SCD in a maternity context and, given the increasing numbers of women with SCD (or SCT) reaching reproductive age, there is a need to shine a light on this issue to raise awareness with a view to improving services for this population of women. This is one of the main reasons for conducting this review. We hope this addresses your concern but we would be happy to take further guidance if required.
