# Supplementary material for: Sickle cell disease and maternity care: A qualitative synthesis of women’s and health providers experiences
Source: PLoS One. 2026 Jul 13;21(7):e0352992. doi: 10.1371/journal.pone.0352992 (PMC13362093; doi:10.1371/journal.pone.0352992)
Supplement: Attachment — Submitted filename: Response to Reviewers 08.09.25 KF.docx [file pone.0352992.s009.docx]

**PONE-D-25-01416R1**

**Sickle cell disease and maternity care: A qualitative synthesis of women’s and health providers experiences.**

**Response to Reviewers**

Many thanks for taking the time to give our article your careful consideration. Comments by the editor and previous reviewers (1 & 2) have been addressed in a previous rebuttal letter and our understanding is that these have been agreed by reviewers 1 & 2. This rebuttal letter addresses comments raised by reviewer 3 and our detailed responses to these queries are outlined below (in red font).

Comments to the Author

1. If the authors have adequately addressed your comments raised in a previous round of review and you feel that this manuscript is now acceptable for publication, you may indicate that here to bypass the “Comments to the Author” section, enter your conflict of interest statement in the “Confidential to Editor” section, and submit your "Accept" recommendation.

Reviewer #1: All comments have been addressed

Reviewer #2: All comments have been addressed

Reviewer #3: (No Response)

2. Is the manuscript technically sound, and do the data support the conclusions?

The manuscript must describe a technically sound piece of scientific research with data that supports the conclusions. Experiments must have been conducted rigorously, with appropriate controls, replication, and sample sizes. The conclusions must be drawn appropriately based on the data presented.

Reviewer #1: No

Reviewer #2: Yes

Reviewer #3: Yes

3. Has the statistical analysis been performed appropriately and rigorously?

Reviewer #1: No

Reviewer #2: Yes

Reviewer #3: N/A

4. Have the authors made all data underlying the findings in their manuscript fully available?

The [PLOS Data policy](https://eur01.safelinks.protection.outlook.com/?url=http%3A%2F%2Ftrack.editorialmanager.com%2FCL0%2Fhttp%3A%252F%252Fwww.plosone.org%252Fstatic%252Fpolicies.action%2523sharing%2F1%2F010f01990d8d24df-c848ce26-bc6a-485d-9548-cc8ff07a79ab-000000%2Fw40LPyqvApQ9kSixNY0XBrLTo4Sqn9301v3xMGYPiig%3D227&data=05%7C02%7CKWFinlayson1%40lancashire.ac.uk%7Ca7a3df89323143202f4308ddea9751fe%7Cebf69982036b4cc4b2027aeb194c5065%7C0%7C0%7C638924657826491431%7CUnknown%7CTWFpbGZsb3d8eyJFbXB0eU1hcGkiOnRydWUsIlYiOiIwLjAuMDAwMCIsIlAiOiJXaW4zMiIsIkFOIjoiTWFpbCIsIldUIjoyfQ%3D%3D%7C0%7C%7C%7C&sdata=KUp6sL1gIOLMsDkOfHps9VsV%2Bp%2B1HR5Tsero5%2BuHndY%3D&reserved=0) requires authors to make all data underlying the findings described in their manuscript fully available without restriction, with rare exception (please refer to the Data Availability Statement in the manuscript PDF file). The data should be provided as part of the manuscript or its supporting information, or deposited to a public repository. For example, in addition to summary statistics, the data points behind means, medians and variance measures should be available. If there are restrictions on publicly sharing data—e.g. participant privacy or use of data from a third party—those must be specified.

Reviewer #1: No

Reviewer #2: Yes

Reviewer #3: Yes

5. Is the manuscript presented in an intelligible fashion and written in standard English?

PLOS ONE does not copyedit accepted manuscripts, so the language in submitted articles must be clear, correct, and unambiguous. Any typographical or grammatical errors should be corrected at revision, so please note any specific errors here.

Reviewer #1: No

Reviewer #2: Yes

Reviewer #3: Yes

6. Review Comments to the Author

Please use the space provided to explain your answers to the questions above. You may also include additional comments for the author, including concerns about dual publication, research ethics, or publication ethics. (Please upload your review as an attachment if it exceeds 20,000 characters)

**Reviewer #1:** (No Response)

**Reviewer #2:** Thank you for addressing the previous reviewers' comments. I would say that I don't have any concern accepting this work for publication.

**Reviewer #3:** I note that the manuscript has already undergone a round of reviews and revisions have been submitted by the authors in response to these. I have been invited to assess the manuscript as a new reviewer,but have considered previous comments and responses in doing so. My additional comments are:

1. The entire manuscript would benefit from editing for syntax as some of the sentences are very long and unwieldy (e.g., the entire Methods section of the abstract is one sentence). In other places, the sentences read repetitive of already provided content. A good proofread for ‘polishing’ and tightening up the text would make for a better flow of text. We appreciate these comments and have split the narrative in the Methods section into two sentences. We have also been through the article again and, where appropriate, have broken up other long sentences (e.g. on pages 5,6,20,23,25,27) and removed repetitive or superfluous text (e.g. on pages 20,21,22,24,25,26)

2. Why is the PRISMA reporting guideline being used when this is a qualitative evidence synthesis. A more appropriate reporting checklist is ENTREQ (Enhancing transparency in reporting the synthesis of qualitative research). Unless the Journal specifically requests PRISMA is used, then I suggest ENTREQ is used instead
<https://bmcmedresmethodol.biomedcentral.com/articles/10.1186/1471-2288-12-181>

Our understanding is that the journal advocates PRISMA reporting guidelines for systematic reviews (quantitative or qualitative). However, we believe our review aligns with the ENTREQ reporting standards and would be happy to include a reference and appendix to support this view if the editor indicates this would be appropriate.

3. Box 1 is not actually a Box, it is a Table (i.e., 2 columns and 2 rows; a Box is simply that with no internal dividers). This should be relabelled as Table 1 and other Table numbers adjusted accordingly. Thank you – this box has been relabelled as Table 1 and the subsequent tables relabelled accordingly.

4. The list of abbreviations appears incomplete, e.g., CNKI (China National Knowledge Infrastructure), MIDIRS (Maternity and Infant Care database), etc., should be included in the list. We have added several more abbreviations to the list on page 4 including those highlighted by the reviewer (above)

5. Where you say ‘20% of abstracts were screened in duplicate to ensure consistency between the reviewers’ what was the outcome of this, i.e., what was the % agreement on these 20%. Also, this appears to contradict with the previous sentence, where it notes two reviewers independently assessed abstracts – do you mean, the 20% were screened as a consistency check, and then both reviewers continued with the remainder. Better clarity is required. Thank you for brining this to our attention. We have now revised this section to aid understanding. The narrative is as follows (see page 9):- ‘*Two members of the review team (KF, GM) independently assessed 50% of the abstracts (each) to determine eligibility against the a priori inclusion/exclusion criteria. Twenty percent of the total number of abstracts were screened in duplicate to ensure consistency between the reviewers and, in the event of a discrepancy, agreement on inclusion was reached by consensus’*. Unfortunately, we can’t include a number for the % agreement of the 20% screened in duplicate as the software we used (Rayyan) automatically updates any ‘conflicts’ once they’ve been resolved.

6. Similarly in quality appraisal, you note a 20% sample were cross-checked, but if both reviewers independently did this, why were all not checked or what was the outcome of the 20% check, and what happened as a result, especially as you then note, a third reviewer examined discrepancies – was this only on the 20%? Clarity is needed here too. Thanks again – the reference to 20% was a mistake and we hope we have now brought some clarity to this section (see page 10):- ‘*Each study was appraised by two authors independently (KF, GM) and then cross-checked by the same authors to ensure consistency. Any studies where there were scoring discrepancies of more than a grade were referred to another author (SD) for moderation. Studies scoring C or higher were included in the final analysis’*. We had no discrepancies of a grade or more and agreed on the study scoring ‘D’ which was excluded at the QA stage and is reported in the Results section.

7. You note the data extraction was carried out by two reviewers – was this independently done and cross-checked or divided equally? Please clarify. Thank you – we have revised the text in this section to better explain the approach (see page 11) - ‘*In a process aligned with thematic synthesis [21] two review authors (KF & GM) worked together to develop codes from the author findings and quote material in the included papers. Using an iterative process the same two authors (KF, GM) then synthesized these codes into descriptive themes (review findings) and reached agreement on the analytical themes following input and discussion from a third author (SD)’.*

.
8. Did the two authors collectively develop the descriptive themes, or independently? please clarify. Similarly, were the GRADE-CERQual assessments done collectively or independently and cross-checked? See above for how the descriptive themes were developed. The GRADE-CERQual assessment exercise was conducted as follows (see page 11):- ‘*KF conducted GRADE-CERQual [22] assessments of the descriptive themes (review findings) and the final grade was reached by consensus following a cross-check of each assessment by GM. Any grade disagreements were referred to SD for moderation’*

9. In the results of search section, a sentence should not begin with a numerical digit, that is, if beginning a sentence with a number, the number should be written out in full (e.g., 620 articles, becomes six hundred and twenty…, etc.). Thank you for pointing this out – we have now written the number out in full

10. I don’t quite understand the inclusion of GRADE-CERQual in Table 2. GRADE-CERQual is applied to discrete findings’ statements, yet here it looks like they are applied to the themes (and unclear if this is the descriptive or analytical theme). Apologies if this is not clear – in accord with the GRADE-CERQual approach and the method selected (Thematic Synthesis) the discrete findings statements are listed as descriptive themes in column 1 of the table; the contributing studies for each descriptive theme are listed in column 2; the GRADE CERQual assessment (for each descriptive theme) are listed in column 3; supporting quotes for each descriptive theme appear in column 4 and the relevant analytical theme is highlighted in column 5.

What I would suggest is to remove any findings from this Table, and report these in a separate Table with the assessment or certainty. For example, under ‘Importance of screening women early’ there appears to be two separate findings’ statements, but one certainty rating, yet when you read the narrative of the analytical theme ‘Beyond the routine of antenatal screening’ there are certainly more than two discrete findings’ statements identifiable and each of these should be extracted out and subjected to GRADE-CERQual. Again, apologies if this is not clear….I’m wondering if there is a fault in the table on the copy sent to you. The ‘Importance of screening women early’ should appear in a distinct row (in bold text) alongside the contributing studies, CERQual grading and supporting quotes for this particular descriptive theme, with the relevant analytical theme it belongs to at the end of the row. The next descriptive theme, ‘Screening as a moral imperative’ should appear in the row below with the contributing studies, CERQual grading, supporting quotes and relevant analytical theme at the end….and so on for all the descriptive themes. The table should make it clear (in column 5) that there are:-

- 3 descriptive themes supporting the analytical theme, ‘Beyond the Routine of Antenatal Screening’.
- 5 descriptive themes supporting the analytical theme, ‘Myths, Misunderstandings and Submissiveness’.
- 4 descriptive themes supporting the analytical theme, ‘Fear and uncertainty in the face of adversity’.
- 2 descriptive themes supporting the analytical theme, ‘The importance of familial and organizational support’. If this is not reflected in the table we can add some text at the beginning of results section to clarify how the descriptive themes inform the analytical themes.

If there is anything else we can do to aid understanding of this table we would be happy to revise.

This goes for the remaining analytical themes and their associated findings. This links to the Appendix, where again it appears the descriptive themes are being subjected to GRADE-CERQual rather than discrete findings. Attention is needed here to adhere to GRADE-CERQual in the assessment of certainty in findings (not themes) and the links between the assessment in the manuscript and that of the evidence profile in the Supp File. Hopefully our revised text about GRADE-CERQual in the Methods section (see comment 8 above) should make it clear that we applied GRADE-CERQual to the descriptive themes, which, in our view, represent ‘discrete findings’ and an appropriate unit of analysis for GRADE-CERQual assessment. As we understand it “*When using CERQual, we assess confidence in each individual review finding. We acknowledge that review findings can be presented in a range of ways (e.g., as themes or theories) as well as at different levels (e.g., descriptive/aggregative and interpretive)” [See link below for details].*

[Applying GRADE-CERQual to qualitative evidence synthesis findings: introduction to the series | Implementation Science | Full Text](https://implementationscience.biomedcentral.com/articles/10.1186/s13012-017-0688-3#citeas)

The final excel page on our S2 appendix represents a Summary of Findings (SoF) table in accord with guidance from the GRADE-CERQual development group – see tables 3 and 4 on the link below.

[Applying GRADE-CERQual to qualitative evidence synthesis findings—paper 2: how to make an overall CERQual assessment of confidence and create a Summary of Qualitative Findings table | Implementation Science | Full Text](https://implementationscience.biomedcentral.com/articles/10.1186/s13012-017-0689-2#Tab1)

By way of context the GRADE-CERQual assessed descriptive themes (review findings) were used to inform the recently published WHO Guidelines on Sickle Cell Disease in Pregnancy, Childbirth and the Interpregnancy period. [WHO recommendations on the management of sickle-cell disease during pregnancy, childbirth and the interpregnancy period](https://www.who.int/publications/i/item/9789240109124). The analytical themes presented in this article represent an additional interpretive step in accord with the method used (thematic synthesis).

Thank you for taking the time to review this article. We hope our responses above provide further clarity and address all of the issues raised.
