# Supplementary material for: Sickle cell disease and maternity care: A qualitative synthesis of women’s and health providers experiences
Source: PLoS One. 2026 Jul 13;21(7):e0352992. doi: 10.1371/journal.pone.0352992 (PMC13362093; doi:10.1371/journal.pone.0352992)
Supplement: Attachment — Submitted filename: REVIEWER COMMENTS.docx [file pone.0352992.s008.docx]

REVIEWER’S COMMENT:

**PONE-D-25-01416
"Sickle cell disease and maternity care: A qualitative synthesis of women’s and health providers experiences.**

This study is a literature review regarding the view and experiences of women with sickle cell trait (SCT) or sickle cell disease (SCD) and their providers during pregnancy. PRISMA method was used. The authors refer this manuscript addresses topics assigned by the World Health Organization. The authors have addressed previous comments.

Further comments:

1. Introduction. 3^rd^ Paragraph is very long. Consider breaking it down to several shorter ones.
2. It is difficult to understand the issues affecting women with SCT. Please make clearer what are differences affecting women who have SCT from those who have SCD.
3. In Table 2, the articles appear to be related to women with SCD or general practitioners. Are any of the studies addressing women with SCT? If so, which ones.
4. Please do not use contractions in the manuscript, such as didn’t. Rather use did not.
5. In the discussion, paragraphs are long. Please divide to make them more readable.
6. Conclusion: Not clear whether there is any conclusion regarding women with SCT.
