# Supplementary material for: Sickle cell disease and maternity care: A qualitative synthesis of women’s and health providers experiences
Source: PLoS One. 2026 Jul 13;21(7):e0352992. doi: 10.1371/journal.pone.0352992 (PMC13362093; doi:10.1371/journal.pone.0352992)
Supplement: Attachment — Submitted filename: Response to Reviewers 22.12.25.docx [file pone.0352992.s010.docx]

**PONE-D-25-01416R1**

**Sickle cell disease and maternity care: A qualitative synthesis of women’s and health providers experiences.**

**Response to Reviewers**

Many thanks for taking the time to give our article your careful consideration. We appreciate the time taken to thoroughly review our manuscript and believe your comments add strength and clarity to our existing narrative. This rebuttal letter addresses comments raised by reviewers 5 and 6 and our detailed responses to these queries are outlined below (in red font).

Reviewer #4: I understand that the manuscript has had three reviewers, and I have read your responses to those comments. Although the journal editor invited me to assess the manuscript as a new reviewer, I have carefully considered the previous reviewers' feedback and the authors' responses as part of my peer review.

I appreciate your efforts in seeking to get this qualitative review on sickle cell disease published. It is crucial to highlight the care and understanding of this disease, particularly in sub-Saharan Africa, where it accounts for approximately 80% of global cases. The article presents a comprehensive and insightful synthesis of qualitative literature affected by this complex and often underrepresented condition.
I commend your rigorous methodological approach and the depth of analysis demonstrated throughout the review. Your work significantly contributes to the existing body of knowledge by illuminating the psychosocial dimensions of sickle cell disease, which are frequently overlooked in clinical discourse. The nuanced understanding presented in the review has important implications for both research and practice, particularly in enhancing person-centred care and informing culturally responsive interventions, especially in LMICs.

I found the review to be both intellectually enriching and practically relevant. It will undoubtedly serve as a valuable resource for healthcare professionals, educators, and policymakers committed to improving the quality of life for individuals living with sickle cell disease.

Once again, thank you for your contribution.

Reviewer #5: I appreciate the authors’ efforts in addressing an important and understudied area within sickle cell disease care. The manuscript has the potential to make a meaningful contribution to the literature. Please find my comments below, which I hope will assist in improving the clarity, rigor, and overall quality of the paper.


Abstract
Methods:
1.I recommend rephrasing the statement “We were looking for…” to a more formal description, such as: “Studies reporting qualitative data from … were included.”

Agreed – we have altered the text in accord with the reviewer’s suggestion

Results
1.Consider including the countries of the included studies in parentheses for added clarity.

Agreed – we have amended the text and added the relevant countries in brackets

Introduction
1.Lines 118 – 120. These statements are not clear. Do you mean the disease considerations/ complications impact views on contraception, etc? Please revise for clarity.

Noted - we have altered the text to aid clarity, *“Due to the risks associated with transferring the sickle cell gene mutation to their children, reproductive decision-making can be a complex process for women with SCD. Their choice of partner as well as views on contraception and antenatal screening all require careful consideration. For some women, in certain contexts, the option to terminate an affected foetus, may lead to particularly difficult and distressing decision-making”.*

Methods
1.Search strategy: Include the full meaning of CNKI at first mention. There are several other instances where acronyms are not fully described initially, or the full description appears after the acronym has already been used. Please review the manuscript and ensure consistency throughout.

There is a glossary on page 4 of the manuscript listing all acronyms and their corresponding wording. However, we have also listed the meaning for CKNI in this section for added clarity. We have also checked the manuscript for any other inconsistencies in our use of acronyms.

2.Study Selection:
a.Is translating text a standard practice in systematic reviews? If so, please provide a supporting reference. Additionally, acknowledge potential limitations of using Google Translate instead of a human translator in the limitations section. Also, indicate which article was translated in the results section (I assume this was Xavier’s article).

As is common practice in systematic reviews with a global scope we did not restrict our searches to studies published in English. Google Translate is a useful tool for translating non-English studies and is particularly efficient with Latin based languages. We have added a reference in the methods section to support the use of Google Translate in this regard*. However, on this occasion, all studies (including those from Brazil) were also available in English so we did not need to use the software for this review. We have added a note in the Results section to make this clear, *“All included studies were available in English so we did not need to use Google Translate software”.*

* Jackson JL, Kuriyama A, Anton A, Choi A, Fournier JP, Geier AK, et al. The Accuracy of Google Translate for Abstracting Data From Non-English-Language Trials for Systematic Reviews. Ann Intern Med. 2019 Nov 5;171(9):677-679. doi: 10.7326/M19-0891. Epub 2019 Jul 30. PMID: 31357212

b.Lines 193-194: This statement is unclear. Consider rephrasing

Apologies for the confusion. This sentence has been changed to read, *“KF and GM selected relevant articles for inclusion with adjudication by a third author (SD) in the event of any disagreement”.*

c.Indicate whether the review was restricted to original research articles. I noticed that a book (Dyson SM) was included; I have further comments on this in the Results section.

Agreed – We have added a sentence in the Study Selection section stating that, *“References for books and book chapters identified from the databases searches were acquired through the University library and assessed for eligibility, i.e. peer reviewed, reporting relevant, empirical, qualitative data”.* We have added a further response to your comments regarding book inclusion in the Results section (under point 3 below).

d.Include a statement about whether studies that included women with SCD or SCT alongside those from the general population were eligible. Some included studies appear to involve participants without SCD or SCT. Also, clarify in the data extraction section how you ensured that only data relevant to the review objectives (i.e., from women with SCD or SCT) were extracted.

Agreed – We have added a sentence under the ‘Criteria for Inclusion’ section stating that, “*Studies reporting on haemoglobinopathy screening alongside the general population were also included provided there was sufficient data from women diagnosed with SCT”.* We have also added a sentence in the ‘Data Extraction and Analysis’ section which reads, *“In studies where the author findings included data from participants not relevant to the research question, i.e. participants who did not have SCD or SCT or had thalassemia rather than SCD, we tried to code and extract data from pertinent sections of the text”.* In addition, we have inserted another sentence in the ‘Results’ section which reads, *“For the six studies focused on screening some of the findings included data from participants relating to thalassemia and some incorporated data from women who had received a negative SCD or SCT screening result. In all of these cases we tried to extract pertinent data from the population of interest by only coding relevant text and ignoring inappropriate quotes”.*

3.Quality Appraisal:
a.The references cited do not match the provided bibliography. Reference 21 in the bibliography is Bulgin et al, not Walsh and Downe. Please review the entire manuscript to ensure all in-text citations match the reference list.

Apologies – we have updated this reference and checked the others

b.Please provide reference(s) for ‘A-D’ scoring.

(Revised) Reference [22] outlines the development of the scoring system and reference [26] illustrates its first use. We have also explained how the scoring system is operationalized in the text and in the S2 Appendix.

c.Line 226 – 229: Include reference(s) for GRADE scoring

(Revised) Reference [24] presents a summary of the GRADE-CERQual scoring system and we have added a further citation to this reference as the reviewer suggests.

There are a series of articles which outline each component of the GRADE-CERQual tool and the associated approaches to scoring. [See this link for further details [Official guidance for applying GRADE-CERQual :: Grade-cerqual](https://www.cerqual.org/official-guidance-for-applying-grade-cerqual/)] We can add these additional references if the reviewer would like. However, we feel that ref [24] encapsulates the tool and the scoring system appropriately and provides an introduction to the additional series of papers should a reader wish to explore further.

Results
1.Line 239 - 240: This statement is not clear. Perhaps you meant to write “did not fulfil the inclusion criteria.”

Apologies – yes, this has been altered accordingly

2.Include citations in the description of all analytic themes. In its current state, readers must refer back to Table 3 to identify sources, which is not ideal.

Agreed – we have added relevant citations to the descriptions of analytic themes

3.Dyson SM: It is generally not standard practice to include books in systematic reviews, and I have several concerns about the inclusion. Was the book peer-reviewed? Can you identify the chapters that provided the data used in the review? Also, do you have references to justify the inclusion of a book? If not, consider excluding the book from your results.

As far as we are aware it is common practice to include books and book chapters in reviews of qualitative literature. As outlined in the Cochrane-Campbell Handbook for Qualitative Evidence Synthesis (2023) [Cochrane-Campbell Handbook for Qualitative Evidence Synthesis | Cochrane](https://www.cochrane.org/authors/handbooks-and-manuals/cochrane-campbell-handbook-qualitative-evidence-synthesis) [chapter 5] and noted in Booth’s methodological review on ‘Searching for qualitative research for inclusion in systematic reviews’ (2016)* “qualitative research is published in books as well as journal articles” and it is important to address this issue when searching for relevant data.

The book by Simon Dyson (2005) included in our review was identified in our database searches. It is a peer reviewed book describing an empirical qualitative study and reports on all aspects of the research process, arguably in far greater detail than one might find in a word-limited journal article. We would also suggest that the identification of particular chapters would not be appropriate in this instance since the full study forms the basis for the book. We hope this addresses your concerns in this regard but would be happy to supply further information if required.

*Booth A. Searching for qualitative research for inclusion in systematic reviews: a structured methodological review. Syst Rev. 2016 May 4;5:74. doi: 10.1186/s13643-016-0249-x. PMID: 27145932; PMCID: PMC4855695

4.Table 2: Consider revising the table for clarity. Combine author and year into one column (e.g., Cox and Beauquier, 2014). Replace the title column with study aims, as this provides a clearer sense of the study’s focus.

Agreed – we have combined the author and date into column 1 of table 2 but we have retained the title as we feel this is an important detail. We have added another column (5) outlining the study aims in accord with the reviewer’s suggestion.

5.Dyson SM et al. 2007: This study included individuals without SCD or SCT. How did you identify observations specific to your population of interest?

See response to point 2(d) in the Study Selection section (above) i.e., *“For the six studies focused on screening some of the findings included data from participants relating to thalassemia and some incorporated data from women who had received a negative SCD or SCT screening result. In all of these cases we tried to extract pertinent data from the population of interest by only coding relevant text and ignoring inappropriate quotes”*.

6.Table 3: The quote provided from reference 34 (Tsianikas et al, 2012) was not from an individual with SCD or SCT. The paper says that P5 was not a carrier. This goes back to my initial comment: how did you ensure that the data used to generate the themes in this review were from the population of interest? Please address this and review all the provided quotes to ensure that they were from the population of interest.

Apologies – we have removed this quote and replaced it with a quote from a participant from the population interest (see revised Table 3). We have also checked all of the additional quotes to ensure they came from relevant participants. In a broader sense, it was difficult to disentangle pertinent data from the findings of several included studies but, as noted previously, we did try to code only those data that reflected findings from our population of interest.

.
7.Table 3: Capitalize the “I” in “intense feelings…”

Thank you for pointing this out – we have changed this “I” to a capital.

Discussion
1.The introduction of the discussion frames findings as relevant only to women with SCD, whereas most included studies focused on individuals with SCT. Please revise to reflect implications for both groups.

Agreed – we have restructured the ‘Discussion’ section to highlight pertinent findings for women diagnosed with SCT. The third paragraph now includes the following additional text, *“For women diagnosed with SCT following antenatal screening appointments our findings illustrate the sense of shock and surprise when an unexpected result is confirmed, especially when the test is presented to them as ‘routine’. Poor understanding of the test and its implications as well as a general lack of knowledge about the genetic origins of the disease highlight notable deficiencies in women’s understanding of SCT and the need for more relevant, understandable, information resources. In addition, our findings suggest that women diagnosed with SCT during antenatal screening would prefer to take the test prior to conception so that reproductive planning and decision-making can occur before they become pregnant. Pre-relationship or pre-marital screening for SCD has been introduced successfully in some European countries (e.g. Greece and Cyprus) though it may not be suitable in all contexts where the identification of carrier status can lead to discrimination or stigmatization* [46]”.

2.There are several grammatical errors in this section; please proofread carefully.

Noted – we have checked the Discussion for grammatical errors and altered text accordingly

3.Second paragraph: You note a lack of knowledge among affected individuals, then discuss implications for the general population. This transition feels abrupt—consider reorganizing for better flow.

Noted – we hope the restructured discussion and additional text on women with SCT addresses this concern and improves the flow of the narrative.

4.Lines 413–415: This statement is unclear. Additionally, the claim that there are no SCD-specific interventions for healthcare providers is incorrect. See Reich et al., 2024 (Pain Management and Nursing). Please revise for accuracy and clarity.

Thank you for providing this additional reference. We have amended the text in this section to include the above reference and have added further narrative to aid clarity, *“Recent reviews of studies exploring the impact of SCD focused educational interventions for healthcare professionals found that provider understanding of the condition could be improved using a variety of educational initiatives [44,45]. Importantly, the authors also identified studies which highlighted improvements in provider attitudes towards people with SCD [44,45]. Although this is encouraging neither review identified any studies conducted in a maternity context where specific knowledge gaps around screening for SCT and the impact of pregnancy on women with SCD (and vice-versa) remain. This highlights the need for the development of bespoke SCD focused training courses for health professionals working with women (and their families) in reproductive and maternal health”.*

Reviewer #6: This study is a literature review regarding the view and experiences of women with sickle cell trait (SCT) or sickle cell disease (SCD) and their providers during pregnancy. PRISMA method was used. The authors refer this manuscript addresses topics assigned by the World Health Organization. The authors have addressed previous comments. It is hard to follow because of long paragraphs. There is lack of clarity between the women with sickle cell disease and those with sickle cell trait.

Noted – we have tried to differentiate between women with SCD and SCT at various stages in the manuscript and have revised our discussion and conclusion sections to aid clarity (e.g. see comment 4 above). It is important to note that we only included studies reporting on women with SCT identified during the antenatal screening process since our focus was on their encounters with maternity services.

Specific comments:
1.Introduction. 3rd Paragraph is very long. Consider breaking it down to several shorter ones.

Agreed – this paragraph has been broken up into several shorter paragraphs.

2.It is difficult to understand the issues affecting women with SCT. Please make clearer what are differences affecting women who have SCT from those who have SCD.

Agreed – we have restructured the discussion and added another paragraph to highlight the key issues faced by women with an SCT diagnosis (see point 4 in response to reviewer 4 above)

3.In Table 2, the articles appear to be related to women with SCD or general practitioners. Are any of the studies addressing women with SCT? If so, which ones.

Noted – in the second paragraph of the Results section we have added a sentence indicating that six of the included studies were focused on SCT screening. In Table 2 it should be clear in the title of the study which ones were focused on SCT screening (see column 1).

4. Please do not use contractions in the manuscript, such as didn’t. Rather use did not.

Noted – we have changed contractions in accord with the reviewer’s suggestion

5.In the discussion, paragraphs are long. Please divide to make them more readable.

Noted - we have checked the paragraphs in the Discussion and, although some are relatively long, they do follow the same theme or line of thinking. However, where this is not the case, we have broken them up into shorter versions in accord with the reviewer’s suggestion

6.Conclusion: Not clear whether there is any conclusion regarding women with SCT.

Agreed – we have restructured the Conclusion into two paragraphs with the first focused on women with SCD and the second on women with SCT (identified via screening). This second paragraph now states, *“To further reduce the impact of SCD in areas of high prevalence, SCT screening programmes offer opportunities for women (and their families) to play an active role in reproductive decision making. Pre-marital or pre-conceptual screening for SCT may be appropriate in some contexts but must be considered alongside the potential for stigmatization. In addition, it is important that screening services are properly resourced and able to provide understandable information in a supportive, non-judgemental environment with well-trained staff sensitive to the needs of women from a variety of different ethnic and cultural backgrounds”*

Thank you for taking the time to review this article. We hope our responses above provide further clarity and address all of the issues raised
