# Supplementary material for: Sickle cell disease and maternity care: A qualitative synthesis of women’s and health providers experiences
Source: PLoS One. 2026 Jul 13;21(7):e0352992. doi: 10.1371/journal.pone.0352992 (PMC13362093; doi:10.1371/journal.pone.0352992)
Supplement: Attachment — Submitted filename: Response to Reviewers 31.03.26 Revised.docx [file pone.0352992.s011.docx]

**PONE-D-25-01416R1**

**Sickle cell disease and maternity care: A qualitative synthesis of women’s and health providers experiences.**

**Response to Reviewers**

Many thanks for taking the time to give this article your careful consideration. We appreciate the time taken to review our manuscript and welcome your comments and suggestions. This rebuttal letter addresses comments raised by reviewer 7 and endorsed by the academic editor. Our responses to these queries are outlined below (in red font).

Reviewer #5: I thank the authors for addressing all the comments. I believe this is very important and needed work, and I thank them once again for taking the initiative to do it.

Thank you…much appreciated

Reviewer #7: Thank you for sharing all the relevant details for the review.
In this article there seems to be 2 major lackings, one is that the language is very unprofessional, refereing to self and using the terms we, our, etc is not the appropriate language for scientific article.

We acknowledge that the language is presented in a more informal style and appreciate that this may not appeal to all reviewers and readers. In accord with the reviewer’s suggestion, we have therefore altered all first-person references (‘we’, ‘our’, etc; ) to the more formal passive style, more common in academic literature.

Secondly, the qualitative analysis should be presented in a tabular or graphical or pictorial form to make sense of the analysis and the process is clear. Although they have included one table representing different responses and analysis but something is missing, it doesnt give a completed sense.

Thank you for bringing this to our attention. As the reviewer indicates we have presented a table (table 3) highlighting the descriptive themes (along with supporting quotes) and the analytical themes, broadly in accord with the methodological requirements associated with thematic analysis. Full details of the data extraction, coding and thematic development are included in the S2 appendix where colour coding was used to map the coded data to the emerging themes. This provides transparency and should allow a reader to follow our analysis in a clear and methodical manner. However, to aid clarity we have also added brief sections of text at the beginning of each analytical theme (in the Results section) highlighting which descriptive themes contribute to each analytical theme. We trust this addresses the reviewers’ comments but if there is anything else we can add to help with understanding please let us know.

All the other comments are well adjusted and revised appropriately. Thank you

Make sure to follow the guidelines for formating and editing os the text and similar font and size should be used.

We have tried to follow the journal formatting and editing guidelines throughout.

Role of Funder statement

As requested in the separate e-mail received on 31.03.2026 we would like to add an additional section of text under the Role of Funder heading which reads,

"The funders had no role in study design, data collection and analysis, decision to publish, or preparation of the manuscript."

Thanks again for taking the time to review this article. We hope our responses above provide further clarity and address the issues raised.
