# Supplementary material for: Sickle cell disease and maternity care: A qualitative synthesis of women’s and health providers experiences
Source: PLoS One. 2026 Jul 13;21(7):e0352992. doi: 10.1371/journal.pone.0352992 (PMC13362093; doi:10.1371/journal.pone.0352992)
Supplement: Peer review history

# Peer review: Decision Letter 0

**DOI:** 10.1371/journal.pone.0352992.r001

**Authors:** Katibe Tugce Temur

16 Feb 2025

PONE-D-25-01416

Sickle cell disease and maternity care: A qualitative synthesis of women’s and health providers experiences.

PLOS ONE

Dear Dr. Finlayson,

Thank you for submitting your manuscript to PLOS ONE. After careful consideration, we feel that it has merit but does not fully meet PLOS ONE’s publication criteria as it currently stands. Therefore, we invite you to submit a revised version of the manuscript that addresses the points raised during the review process.

Please submit your revised manuscript by Apr 02 2025 11:59PM. If you will need more time than this to complete your revisions, please reply to this message or contact the journal office at plosone@plos.org. When you're ready to submit your revision, log on to https://www.editorialmanager.com/pone/ and select the 'Submissions Needing Revision' folder to locate your manuscript file.

Please include the following items when submitting your revised manuscript:

- A rebuttal letter that responds to each point raised by the academic editor and reviewer(s). You should upload this letter as a separate file labeled 'Response to Reviewers'.
- A marked-up copy of your manuscript that highlights changes made to the original version. You should upload this as a separate file labeled 'Revised Manuscript with Track Changes'.
- An unmarked version of your revised paper without tracked changes. You should upload this as a separate file labeled 'Manuscript'.

-->If you would like to make changes to your financial disclosure, please include your updated statement in your cover letter. Guidelines for resubmitting your figure files are available below the reviewer comments at the end of this letter.

If applicable, we recommend that you deposit your laboratory protocols in protocols.io to enhance the reproducibility of your results. Protocols.io assigns your protocol its own identifier (DOI) so that it can be cited independently in the future. For instructions see: https://journals.plos.org/plosone/s/submission-guidelines#loc-laboratory-protocols. Additionally, PLOS ONE offers an option for publishing peer-reviewed Lab Protocol articles, which describe protocols hosted on protocols.io. Read more information on sharing protocols at https://plos.org/protocols?utm_medium=editorial-email&utm_source=authorletters&utm_campaign=protocols.

We look forward to receiving your revised manuscript.

Kind regards,

Katibe Tugce Temur

Academic Editor

PLOS ONE

Journal Requirements:

When submitting your revision, we need you to address these additional requirements.

1. Please ensure that your manuscript meets PLOS ONE's style requirements, including those for file naming. The PLOS ONE style templates can be found at

https://journals.plos.org/plosone/s/file?id=wjVg/PLOSOne_formatting_sample_main_body.pdf and

https://journals.plos.org/plosone/s/file?id=ba62/PLOSOne_formatting_sample_title_authors_affiliations.pdf

2. Please remove all personal information, ensure that the data shared are in accordance with participant consent, and re-upload a fully anonymized data set.

Note: spreadsheet columns with personal information must be removed and not hidden as all hidden columns will appear in the published file.

Additional guidance on preparing raw data for publication can be found in our Data Policy (https://journals.plos.org/plosone/s/data-availability#loc-human-research-participant-data-and-other-sensitive-data) and in the following article: http://www.bmj.com/content/340/bmj.c181.long.

3. As required by our policy on Data Availability, please ensure your manuscript or supplementary information includes the following:

A numbered table of all studies identified in the literature search, including those that were excluded from the analyses.

For every excluded study, the table should list the reason(s) for exclusion.

If any of the included studies are unpublished, include a link (URL) to the primary source or detailed information about how the content can be accessed.

A table of all data extracted from the primary research sources for the systematic review and/or meta-analysis. The table must include the following information for each study:

Name of data extractors and date of data extraction

Confirmation that the study was eligible to be included in the review.

All data extracted from each study for the reported systematic review and/or meta-analysis that would be needed to replicate your analyses.

If data or supporting information were obtained from another source (e.g. correspondence with the author of the original research article), please provide the source of data and dates on which the data/information were obtained by your research group.

If applicable for your analysis, a table showing the completed risk of bias and quality/certainty assessments for each study or outcome.  Please ensure this is provided for each domain or parameter assessed. For example, if you used the Cochrane risk-of-bias tool for randomized trials, provide answers to each of the signalling questions for each study. If you used GRADE to assess certainty of evidence, provide judgements about each of the quality of evidence factor. This should be provided for each outcome.

An explanation of how missing data were handled.

This information can be included in the main text, supplementary information, or relevant data repository. Please note that providing these underlying data is a requirement for publication in this journal, and if these data are not provided your manuscript might be rejected.

4. We note that the grant information you provided in the ‘Funding Information’ and ‘Financial Disclosure’ sections do not match.

When you resubmit, please ensure that you provide the correct grant numbers for the awards you received for your study in the ‘Funding Information’ section.

5. Please review your reference list to ensure that it is complete and correct. If you have cited papers that have been retracted, please include the rationale for doing so in the manuscript text, or remove these references and replace them with relevant current references. Any changes to the reference list should be mentioned in the rebuttal letter that accompanies your revised manuscript. If you need to cite a retracted article, indicate the article’s retracted status in the References list and also include a citation and full reference for the retraction notice.

Additional Editor Comments:

Dear Author,

Thank you for submitting your manuscript for evaluation. However, in order to strengthen and clarify your manuscript, the following revisions are recommended:

Introduction Section:

You should clearly emphasize the necessity of your study and explicitly address the research gap to make the introduction more focused.

Methods Section:

Inclusion and exclusion criteria should be described in more detail. This is crucial for ensuring the reproducibility of the study.

Discussion Section:

The discussion should be concise, and redundant points should be removed.

Most of the included studies originate from the United Kingdom and Brazil. This may limit the generalizability of the findings to other regions, particularly to low-income countries (LICs), where SCD is more prevalent. This limitation should be explicitly acknowledged, and the potential variations in findings across different contexts should be discussed.

Only three studies focusing on healthcare providers are included, all of which are from the United Kingdom. This limits the understanding of healthcare providers' experiences in different healthcare systems. This limitation should be addressed, and future research directions should be proposed.

The discussion should conclude with recommendations for future studies.

Abbreviations Table:

To enhance readability, please provide a table listing the abbreviations used in the manuscript.

Limitations Section:

A separate section should be added to discuss the limitations of the study in greater detail.

References:

Since most of the references are older than five years, it is recommended to update some of them with more recent literature.

Additionally, we encourage you to review your manuscript for compliance with PLOS ONE formatting and writing guidelines. A general revision regarding language and clarity would also be beneficial. Please submit a revised version with the implemented changes clearly indicated.

Best regards,

Editor

[Note: HTML markup is below. Please do not edit.]

Reviewers' comments:

Reviewer's Responses to Questions

**Comments to the Author**

1. Is the manuscript technically sound, and do the data support the conclusions?

The manuscript must describe a technically sound piece of scientific research with data that supports the conclusions. Experiments must have been conducted rigorously, with appropriate controls, replication, and sample sizes. The conclusions must be drawn appropriately based on the data presented.

Reviewer #1: Yes

Reviewer #2: Yes

**********

2. Has the statistical analysis been performed appropriately and rigorously?

Reviewer #1: Yes

Reviewer #2: N/A

**********

3. Have the authors made all data underlying the findings in their manuscript fully available?

The PLOS Data policy requires authors to make all data underlying the findings described in their manuscript fully available without restriction, with rare exception (please refer to the Data Availability Statement in the manuscript PDF file). The data should be provided as part of the manuscript or its supporting information, or deposited to a public repository. For example, in addition to summary statistics, the data points behind means, medians and variance measures should be available. If there are restrictions on publicly sharing data—e.g. participant privacy or use of data from a third party—those must be specified.

Reviewer #1: No

Reviewer #2: Yes

**********

4. Is the manuscript presented in an intelligible fashion and written in standard English?

PLOS ONE does not copyedit accepted manuscripts, so the language in submitted articles must be clear, correct, and unambiguous. Any typographical or grammatical errors should be corrected at revision, so please note any specific errors here.

Reviewer #1: Yes

Reviewer #2: Yes

**********

5. Review Comments to the Author

Please use the space provided to explain your answers to the questions above. You may also include additional comments for the author, including concerns about dual publication, research ethics, or publication ethics. (Please upload your review as an attachment if it exceeds 20,000 characters)

Reviewer #1: Dear Author

Thank you for submitting your article for review

Your study is very valuable

The following comments are provided to improve the research:

In the introduction section, the necessity of the work should be clearly stated

In the methods section, the inclusion and exclusion criteria of the study should be more precisely stated.

The discussion should be strengthened.

At the end of the discussion, suggestions for future studies should be mentioned.

Thank you

Reviewer #2: Thank you for your qualitative paper on: Sickle Cell Disease and Maternity Care: A Qualitative Synthesis of Women’s and Health Providers’ Experiences

Please find my comments

1- Introduction could be focused, addressing the research gap as well.

2- Discussion needs to be concise, some points are repeated .

3- The majority of the included studies are from the UK and Brazil, which limits the transferability of the findings to other regions, particularly low-income countries (LICs) where SCD is highly prevalent. The authors should acknowledge this limitation more explicitly and discuss how the findings might differ in other contexts.

4- The manuscript includes only three studies focusing on healthcare providers, all from the UK. This limits the understanding of provider experiences in different healthcare systems. The authors should discuss this limitation and suggest areas for future research.

5- Please provide a table for abbreviations.

6- Please provide a section for limitations.

7- Please update some of your references, as most of your references are more than 5 years outdated.

**********

6. PLOS authors have the option to publish the peer review history of their article (what does this mean?). If published, this will include your full peer review and any attached files.

If you choose “no”, your identity will remain anonymous but your review may still be made public.

**Do you want your identity to be public for this peer review?**  For information about this choice, including consent withdrawal, please see our Privacy Policy.

Reviewer #1: No

Reviewer #2: No

**********

[NOTE: If reviewer comments were submitted as an attachment file, they will be attached to this email and accessible via the submission site. Please log into your account, locate the manuscript record, and check for the action link "View Attachments". If this link does not appear, there are no attachment files.]

While revising your submission, please upload your figure files to the Preflight Analysis and Conversion Engine (PACE) digital diagnostic tool, https://pacev2.apexcovantage.com/. PACE helps ensure that figures meet PLOS requirements. To use PACE, you must first register as a user. Registration is free. Then, login and navigate to the UPLOAD tab, where you will find detailed instructions on how to use the tool. If you encounter any issues or have any questions when using PACE, please email PLOS at figures@plos.org. Please note that Supporting Information files do not need this step.

# Author comment: Author response to Decision Letter 1

**DOI:** 10.1371/journal.pone.0352992.r002

1 Apr 2025

PONE-D-25-01416

Sickle cell disease and maternity care: A qualitative synthesis of women’s and health providers experiences.

Response to Reviewers

Many thanks for taking the time to give our article your careful consideration. Detailed responses to all queries and concerns raised by the editor and reviewers are outlined below (in red font).

When submitting your revision, we need you to address these additional requirements.

1. Please ensure that your manuscript meets PLOS ONE's style requirements, including those for file naming. The PLOS ONE style templates can be found at

https://journals.plos.org/plosone/s/file?id=wjVg/PLOSOne_formatting_sample_main_body.pdf and

https://journals.plos.org/plosone/s/file?id=ba62/PLOSOne_formatting_sample_title_authors_affiliations.pdf

We have checked this guidance and believe our manuscript meets with journal requirements but are happy to accept further advice if this is not the case

2. Please remove all personal information, ensure that the data shared are in accordance with participant consent, and re-upload a fully anonymized data set.

We believe all data sets are fully anonymized. This is a review of existing, published, qualitative articles and not an empirical study so, as far as we are aware, participant consent is not required

Note: spreadsheet columns with personal information must be removed and not hidden as all hidden columns will appear in the published file.

We believe all spreadsheet columns are ‘clean’ and do not contain any personal information

3. As required by our policy on Data Availability, please ensure your manuscript or supplementary information includes the following:

A numbered table of all studies identified in the literature search, including those that were excluded from the analyses.

We have added an additional supplementary table (labelled S2 Appendix) listing all of the 47 studies identified in our searches (at full text stage) including the author(s), title, date of publication, country (and income level), population under investigation, whether the study was excluded or included and, where relevant, the reason for exclusion.

For every excluded study, the table should list the reason(s) for exclusion.

Reasons for exclusion have been added in the S2 Appendix, as noted above.

If any of the included studies are unpublished, include a link (URL) to the primary source or detailed information about how the content can be accessed.

All of the included studies are published and the references provided in the reference section. Findings from one study (Ref no 28) were extracted from a book and a hyperlink to the book source has been added to this reference. Ethnicity and screening for sickle cell/thalassaemia : lessons for practice from the voices of experience / Simon M. Dyson. | Wellcome Collection

A table of all data extracted from the primary research sources for the systematic review and/or meta-analysis. The table must include the following information for each study:

All of the information relating to the studies included in this review is located in the Excel spreadsheet labelled as ‘S3 Appendix’. This includes details of the study characteristics (page 1); a detailed quality appraisal of each study (page 2); the coding and data extraction of findings from each study (page 3); the final themes (page 4) and a GRADE CERQual appraisal (including a level of confidence grading) of the final themes on page 5. We believe this file makes our approaches towards data extraction, analysis and synthesis robust and transparent.

Name of data extractors and date of data extraction

We have added the names of the data extractors and the dates when data was extracted to the ‘Data Extraction and Analysis’ section of the manuscript on page 11.

Confirmation that the study was eligible to be included in the review.

This information is available in the supplementary files, S2 and S3 Appendix

All data extracted from each study for the reported systematic review and/or meta-analysis that would be needed to replicate your analyses.

From a qualitative perspective this information is reported clearly and transparently on pages 3 and 4 of the S3 Appendix

If data or supporting information were obtained from another source (e.g. correspondence with the author of the original research article), please provide the source of data and dates on which the data/information were obtained by your research group.

We didn’t retrieve any data from additional sources.

If applicable for your analysis, a table showing the completed risk of bias and quality/certainty assessments for each study or outcome. Please ensure this is provided for each domain or parameter assessed. For example, if you used the Cochrane risk-of-bias tool for randomized trials, provide answers to each of the signalling questions for each study. If you used GRADE to assess certainty of evidence, provide judgements about each of the quality of evidence factor. This should be provided for each outcome.

Since this is a qualitative systematic review we are not reporting on pre-determined quantitative outcomes. We used the GRADE-CERQual tool (described on pages 8-9 in the manuscript) to assess confidence (high, moderate, low or very low) in the review findings in accord with Cochrane approaches to the reporting of qualitative data from these types of reviews. The GRADE-CERQual assessments for each review finding (theme) are highlighted in Table 1 and the details of how these grades were achieved is displayed on page 5 of the S3 Appendix.

An explanation of how missing data were handled.

As far as we are aware there is no missing data.

This information can be included in the main text, supplementary information, or relevant data repository. Please note that providing these underlying data is a requirement for publication in this journal, and if these data are not provided your manuscript might be rejected.

See notes above – we believe we have provided all underlying data

4. We note that the grant information you provided in the ‘Funding Information’ and ‘Financial Disclosure’ sections do not match.

When you resubmit, please ensure that you provide the correct grant numbers for the awards you received for your study in the ‘Funding Information’ section.

We have provided a financial statement at the beginning of this letter:-

“This work was commissioned to the University of Central Lancashire by the UNDP/ UNFPA/UNICEF/WHO/World Bank Special Programme of Research, Development and Research Training in Human Reproduction (HRP), a cosponsored program executed by the World Health Organization (WHO)”

5. Please review your reference list to ensure that it is complete and correct. If you have cited papers that have been retracted, please include the rationale for doing so in the manuscript text, or remove these references and replace them with relevant current references.

As far as we are aware we have not cited any publications that have been retracted.

Any changes to the reference list should be mentioned in the rebuttal letter that accompanies your revised manuscript. If you need to cite a retracted article, indicate the article’s retracted status in the References list and also include a citation and full reference for the retraction notice.

Additional Editor Comments:

Dear Author,

Thank you for submitting your manuscript for evaluation. However, in order to strengthen and clarify your manuscript, the following revisions are recommended:

Introduction Section:

You should clearly emphasize the necessity of your study and explicitly address the research gap to make the introduction more focused.

We have removed some superfluous text from the introduction, added some text to highlight the research gap and made the objectives of the study more clear (see pages 4 & 5 in the introduction section).

Methods Section:

Inclusion and exclusion criteria should be described in more detail. This is crucial for ensuring the reproducibility of the study.

In addition to the text under ‘Criteria for Inclusion’ on page 6 we have added a box (Box 1) listing the inclusion and exclusion criteria (see pages 7 & 8)

Discussion Section:

The discussion should be concise, and redundant points should be removed.

We have removed and/or summarized sections of text in the discussion to make it more concise (see pages 23-27)

Most of the included studies originate from the United Kingdom and Brazil. This may limit the generalizability of the findings to other regions, particularly to low-income countries (LICs), where SCD is more prevalent. This limitation should be explicitly acknowledged, and the potential variations in findings across different contexts should be discussed.

We have added a sub-title ‘Limitations of the Review’ on page 27 where the issues of generalizability and transferability are explicitly acknowledged.

Only three studies focusing on healthcare providers are included, all of which are from the United Kingdom. This limits the understanding of healthcare providers' experiences in different healthcare systems. This limitation should be addressed, and future research directions should be proposed.

This issue is acknowledged and addressed under the new sub-heading, ‘Limitations of the Review’ on page 27

The discussion should conclude with recommendations for future studies.

We have added a paragraph at the end of the discussion section with recommendations for future studies

Abbreviations Table:

To enhance readability, please provide a table listing the abbreviations used in the manuscript.

We have added a List of Abbreviations after the Abstract

Limitations Section:

A separate section should be added to discuss the limitations of the study in greater detail.

As noted above we have added a Limitations section on page 27

References:

Since most of the references are older than five years, it is recommended to update some of them with more recent literature.

As outlined in our ‘Criteria for Inclusion’ (page 8) our searches were conducted during the period 2000-2024 to ensure our studies captured the views of the current generation of women (and health professionals). 10 of our 11 included studies (Ref’s 27-37) were conducted prior to 2019 with the oldest conducted in 2005 so this is something we can’t change. However, we appreciate your comment and have added a sentence to the ‘Limitations’ section to reflect this concern. With regard to the supporting references we have been through them all and note there are a few older ones (pre 2010) in the methods section which again we can’t change because they cite methodological papers as ‘benchmark studies’ for a particular methodology or approach to analysis/synthesis, i.e. refs 22,23 & 25. Of the remaining references most are current or refer to a specific (and relevant) set of data which, as far as we are aware, has not been updated recently (e.g. ref’s 2 & 6). In a general sense there are a limited number of studies exploring SCD in a maternity context and, given the increasing numbers of women with SCD (or SCT) reaching reproductive age, there is a need to shine a light on this issue to raise awareness with a view to improving services for this population of women. This is one of the main reasons for conducting this review. We hope this addresses your concerns but we would be happy to take further guidance if required.

Additionally, we encourage you to review your manuscript for compliance with PLOS ONE formatting and writing guidelines. A general revision regarding language and clarity would also be beneficial. Please submit a revised version with the implemented changes clearly indicated.

We believe this review meets PLOS ONE formatting and writing guidelines and we hope we have addressed any concerns in the revised version

Reviewers' comments:

Reviewer's Responses to Questions

Comments to the Author

1. Is the manuscript technically sound, and do the data support the conclusions?

The manuscript must describe a technically sound piece of scientific research with data that supports the conclusions. Experiments must have been conducted rigorously, with appropriate controls, replication, and sample sizes. The conclusions must be drawn appropriately based on the data presented.

Reviewer #1: Yes

Reviewer #2: Yes

________________________________________

2. Has the statistical analysis been performed appropriately and rigorously?

Reviewer #1: Yes

Reviewer #2: N/A

________________________________________

3. Have the authors made all data underlying the findings in their manuscript fully available?

The PLOS Data policy requires authors to make all data underlying the findings described in their manuscript fully available without restriction, with rare exception (please refer to the Data Availability Statement in the manuscript PDF file). The data should be provided as part of the manuscript or its supporting information, or deposited to a public repository. For example, in addition to summary statistics, the data points behind means, medians and variance measures should be available. If there are restrictions on publicly sharing data—e.g. participant privacy or use of data from a third party—those must be specified.

Reviewer #1: No

We believe we have supplied all supporting data – either in the manuscript or in the S1, S2 or S3 appendices

Reviewer #2: Yes

________________________________________

4. Is the manuscript presented in an intelligible fashion and written in standard English?

PLOS ONE does not copyedit accepted manuscripts, so the language in submitted articles must be clear, correct, and unambiguous. Any typographical or grammatical errors should be corrected at revision, so please note any specific errors here.

Reviewer #1: Yes

Reviewer #2: Yes

________________________________________

5. Review Comments to the Author

Please use the space provided to explain your answers to the questions above. You may also include additional comments for the author, including concerns about dual publication, research ethics, or publication ethics. (Please upload your review as an attachment if it exceeds 20,000 characters)

Reviewer #1: Dear Author

Thank you for submitting your article for review

Your study is very valuable. Thank you.

The following comments are provided to improve the research:

In the introduction section, the necessity of the work should be clearly stated.

We have amended the Introduction section to highlight the evidence gap more clearly and have outlined the objectives of the study towards the end of the Introduction (see pages 5 & 6)

In the methods section, the inclusion and exclusion criteria of the study should be more precisely stated.

We have added a box (Box 1) where the inclusion and exclusion criteria are clearly stated (see pages 8 & 9)

The discussion should be strengthened.

We have amended the Discussion section by removing some superfluous text and clearly stating the limitations of the review under a separate heading.

At the end of the discussion, suggestions for future studies should be mentioned.

We have also added some text at the end of the discussion highlighting some areas for future research (page 29).

Thank you

Reviewer #2: Thank you for your qualitative paper on: Sickle Cell Disease and Maternity Care: A Qualitative Synthesis of Women’s and Health Providers’ Experiences

Please find my comments

1- Introduction could be focused, addressing the research gap as well.

We have amended the Introduction section to highlight the evidence gap more clearly and have outlined the objectives of the study towards the end of the Introduction (see pages 5 & 6)

2- Discussion needs to be concise, some points are repeated .

We have amended the Discussion, removed some superfluous text and tried to remove any repetitive narrative from this section. We hope it is now more concise.

3- The majority of the included studies are from the UK and Brazil, which limits the transferability of the findings to other regions, particularly low-income countries (LICs) where SCD is highly prevalent. The authors should acknowledge this limitation more explicitly and d

Attachment Submitted filename: Response to Reviewers.docx

# Peer review: Decision Letter 1

**DOI:** 10.1371/journal.pone.0352992.r003

**Authors:** Katibe Tugce Temur, Abiola Olaleye

2 Sep 2025

PONE-D-25-01416R1

Sickle cell disease and maternity care: A qualitative synthesis of women’s and health providers experiences.

PLOS ONE

Dear Dr. Finlayson,

Thank you for submitting your manuscript to PLOS ONE. After careful consideration, we feel that it has merit but does not fully meet PLOS ONE’s publication criteria as it currently stands. Therefore, we invite you to submit a revised version of the manuscript that addresses the points raised during the review process.

**Kindly the comments of the reviewers are well addressed.**

Please submit your revised manuscript by Oct 17 2025 11:59PM. If you will need more time than this to complete your revisions, please reply to this message or contact the journal office at plosone@plos.org. When you're ready to submit your revision, log on to https://www.editorialmanager.com/pone/ and select the 'Submissions Needing Revision' folder to locate your manuscript file.

Please include the following items when submitting your revised manuscript:

- A rebuttal letter that responds to each point raised by the academic editor and reviewer(s). You should upload this letter as a separate file labeled 'Response to Reviewers'.
- A marked-up copy of your manuscript that highlights changes made to the original version. You should upload this as a separate file labeled 'Revised Manuscript with Track Changes'.
- An unmarked version of your revised paper without tracked changes. You should upload this as a separate file labeled 'Manuscript'.

-->If you would like to make changes to your financial disclosure, please include your updated statement in your cover letter. Guidelines for resubmitting your figure files are available below the reviewer comments at the end of this letter.

If applicable, we recommend that you deposit your laboratory protocols in protocols.io to enhance the reproducibility of your results. Protocols.io assigns your protocol its own identifier (DOI) so that it can be cited independently in the future. For instructions see: https://journals.plos.org/plosone/s/submission-guidelines#loc-laboratory-protocols. Additionally, PLOS ONE offers an option for publishing peer-reviewed Lab Protocol articles, which describe protocols hosted on protocols.io. Read more information on sharing protocols at https://plos.org/protocols?utm_medium=editorial-email&utm_source=authorletters&utm_campaign=protocols.

We look forward to receiving your revised manuscript.

Kind regards,

Abiola O. Olaleye, MBBS, MPH, FWACP

Academic Editor

PLOS ONE

**Journal Requirements:**

1. If the reviewer comments include a recommendation to cite specific previously published works, please review and evaluate these publications to determine whether they are relevant and should be cited. There is no requirement to cite these works unless the editor has indicated otherwise.

2. Please review your reference list to ensure that it is complete and correct. If you have cited papers that have been retracted, please include the rationale for doing so in the manuscript text, or remove these references and replace them with relevant current references. Any changes to the reference list should be mentioned in the rebuttal letter that accompanies your revised manuscript. If you need to cite a retracted article, indicate the article’s retracted status in the References list and also include a citation and full reference for the retraction notice.

[Note: HTML markup is below. Please do not edit.]

Reviewers' comments:

Reviewer's Responses to Questions

**Comments to the Author**

1. If the authors have adequately addressed your comments raised in a previous round of review and you feel that this manuscript is now acceptable for publication, you may indicate that here to bypass the “Comments to the Author” section, enter your conflict of interest statement in the “Confidential to Editor” section, and submit your "Accept" recommendation.

Reviewer #1: All comments have been addressed

Reviewer #2: All comments have been addressed

Reviewer #3: (No Response)

**********

2. Is the manuscript technically sound, and do the data support the conclusions?

The manuscript must describe a technically sound piece of scientific research with data that supports the conclusions. Experiments must have been conducted rigorously, with appropriate controls, replication, and sample sizes. The conclusions must be drawn appropriately based on the data presented.

Reviewer #1: No

Reviewer #2: Yes

Reviewer #3: Yes

**********

3. Has the statistical analysis been performed appropriately and rigorously?

Reviewer #1: No

Reviewer #2: Yes

Reviewer #3: N/A

**********

4. Have the authors made all data underlying the findings in their manuscript fully available?

The PLOS Data policy requires authors to make all data underlying the findings described in their manuscript fully available without restriction, with rare exception (please refer to the Data Availability Statement in the manuscript PDF file). The data should be provided as part of the manuscript or its supporting information, or deposited to a public repository. For example, in addition to summary statistics, the data points behind means, medians and variance measures should be available. If there are restrictions on publicly sharing data—e.g. participant privacy or use of data from a third party—those must be specified.

Reviewer #1: No

Reviewer #2: Yes

Reviewer #3: Yes

**********

5. Is the manuscript presented in an intelligible fashion and written in standard English?

PLOS ONE does not copyedit accepted manuscripts, so the language in submitted articles must be clear, correct, and unambiguous. Any typographical or grammatical errors should be corrected at revision, so please note any specific errors here.

Reviewer #1: No

Reviewer #2: Yes

Reviewer #3: Yes

**********

6. Review Comments to the Author

Please use the space provided to explain your answers to the questions above. You may also include additional comments for the author, including concerns about dual publication, research ethics, or publication ethics. (Please upload your review as an attachment if it exceeds 20,000 characters)

**Reviewer #1:**  (No Response)

**Reviewer #2:** Thank you for addressing the previous reviewers' comments. I would say that I don't have any concern accepting this work for publication.

**Reviewer #3:** I note that the manuscript has already undergone a round of reviews and revisions have been submitted by the authors in response to these. I have been invited to assess the manuscript as a new reviewer, but have considered previous comments and responses in doing so. My additional comments are:

1. The entire manuscript would benefit from editing for syntax as some of the sentences are very long and unwieldy (e.g., the entire Methods section of the abstract is one sentence). In other places, the sentences read repetitive of already provided content. A good proofread for ‘polishing’ and tightening up the text would make for a better flow of text.

2. Why is the PRISMA reporting guideline being used when this is a qualitative evidence synthesis. A more appropriate reporting checklist is ENTREQ (Enhancing transparency in reporting the synthesis of qualitative research). Unless the Journal specifically requests PRISMA is used, then I suggest ENTREQ is used instead

https://bmcmedresmethodol.biomedcentral.com/articles/10.1186/1471-2288-12-181

3. Box 1 is not actually a Box, it is a Table (i.e., 2 columns and 2 rows; a Box is simply that with no internal dividers). This should be relabelled as Table 1 and other Table numbers adjusted accordingly.

4. The list of abbreviations appears incomplete, e.g., CNKI (China National Knowledge Infrastructure), MIDIRS (Maternity and Infant Care database), etc., should be included in the list

5. Where you say ‘20% of abstracts were screened in duplicate to ensure consistency between the reviewers’ what was the outcome of this, i.e., what was the % agreement on these 20%. Also, this appears to contradict with the previous sentence, where it notes two reviewers independently assessed abstracts – do you mean, the 20% were screened as a consistency check, and then both reviewers continued with the remainder. Better clarity is required

6. Similarly in quality appraisal, you note a 20% sample were cross-checked, but if both reviewers independently did this, why were all not checked or what was the outcome of the 20% check, and what happened as a result, especially as you then note, a third reviewer examined discrepancies – was this only on the 20%? Clarity is needed here too.

7. You note the data extraction was carried out by two reviewers – was this independently done and cross-checked or divided equally? Please clarify.

8. Did the two authors collectively develop the descriptive themes, or independently? please clarify. Similarly, were the GRADE-CERQual assessments done collectively or independently and cross-checked?

9. In the results of search section, a sentence should not begin with a numerical digit, that is, if beginning a sentence with a number, the number should be written out in full (e.g., 620 articles, becomes six hundred and twenty…, etc.).

10. I don’t quite understand the inclusion of GRADE-CERQual in Table 2. GRADE-CERQual is applied to discrete findings’ statements, yet here it looks like they are applied to the themes (and unclear if this is the descriptive or analytical theme). What I would suggest is to remove any findings from this Table, and report these in a separate Table with the assessment or certainty. For example, under ‘Importance of screening women early’ there appears to be two separate findings’ statements, but one certainty rating, yet when you read the narrative of the analytical theme ‘Beyond the routine of antenatal screening’ there are certainly more than two discrete findings’ statements identifiable and each of these should be extracted out and subjected to GRADE-CERQual. This goes for the remaining analytical themes and their associated findings. This links to the Appendix, where again it appears the descriptive themes are being subjected to GRADE-CERQual rather than discrete findings. Attention is needed here to adhere to GRADE-CERQual in the assessment of certainty in findings (not themes) and the links between the assessment in the manuscript and that of the evidence profile in the Supp File.

**********

7. PLOS authors have the option to publish the peer review history of their article (what does this mean?). If published, this will include your full peer review and any attached files.

If you choose “no”, your identity will remain anonymous but your review may still be made public.

**Do you want your identity to be public for this peer review?**  For information about this choice, including consent withdrawal, please see our Privacy Policy.

Reviewer #1: No

Reviewer #2: No

Reviewer #3: No

**********

[NOTE: If reviewer comments were submitted as an attachment file, they will be attached to this email and accessible via the submission site. Please log into your account, locate the manuscript record, and check for the action link "View Attachments". If this link does not appear, there are no attachment files.]

While revising your submission, please upload your figure files to the Preflight Analysis and Conversion Engine (PACE) digital diagnostic tool, https://pacev2.apexcovantage.com/. PACE helps ensure that figures meet PLOS requirements. To use PACE, you must first register as a user. Registration is free. Then, login and navigate to the UPLOAD tab, where you will find detailed instructions on how to use the tool. If you encounter any issues or have any questions when using PACE, please email PLOS at figures@plos.org. Please note that Supporting Information files do not need this step.

# Author comment: Author response to Decision Letter 2

**DOI:** 10.1371/journal.pone.0352992.r004

10 Sep 2025

10th September 2025.

Our responses to Reviewer 3's comments are highlighted below and in the 'Response to Reviewers' letter accompanying this submission.

1. The entire manuscript would benefit from editing for syntax as some of the sentences are very long and unwieldy (e.g., the entire Methods section of the abstract is one sentence). In other places, the sentences read repetitive of already provided content. A good proofread for ‘polishing’ and tightening up the text would make for a better flow of text.

We appreciate these comments and have split the narrative in the Methods section into two sentences. We have also been through the article again and, where appropriate, have broken up other long sentences (e.g. on pages 5,6,20,23,25,27) and removed repetitive or superfluous text (e.g. on pages 20,21,22,24,25,26)

2. Why is the PRISMA reporting guideline being used when this is a qualitative evidence synthesis. A more appropriate reporting checklist is ENTREQ (Enhancing transparency in reporting the synthesis of qualitative research). Unless the Journal specifically requests PRISMA is used, then I suggest ENTREQ is used instead

https://bmcmedresmethodol.biomedcentral.com/articles/10.1186/1471-2288-12-181

Our understanding is that the journal advocates PRISMA reporting guidelines for systematic reviews (quantitative or qualitative). However, we believe our review aligns with the ENTREQ reporting standards and would be happy to include a reference and appendix to support this view if the editor indicates this would be appropriate.

3. Box 1 is not actually a Box, it is a Table (i.e., 2 columns and 2 rows; a Box is simply that with no internal dividers). This should be relabelled as Table 1 and other Table numbers adjusted accordingly.

Thank you – this box has been relabelled as Table 1 and the subsequent tables relabelled accordingly.

4. The list of abbreviations appears incomplete, e.g., CNKI (China National Knowledge Infrastructure), MIDIRS (Maternity and Infant Care database), etc., should be included in the list.

We have added several more abbreviations to the list on page 4 including those highlighted by the reviewer (above)

5. Where you say ‘20% of abstracts were screened in duplicate to ensure consistency between the reviewers’ what was the outcome of this, i.e., what was the % agreement on these 20%. Also, this appears to contradict with the previous sentence, where it notes two reviewers independently assessed abstracts – do you mean, the 20% were screened as a consistency check, and then both reviewers continued with the remainder. Better clarity is required.

Thank you for brining this to our attention. We have now revised this section to aid understanding. The narrative is as follows (see page 9):- ‘Two members of the review team (KF, GM) independently assessed 50% of the abstracts (each) to determine eligibility against the a priori inclusion/exclusion criteria. Twenty percent of the total number of abstracts were screened in duplicate to ensure consistency between the reviewers and, in the event of a discrepancy, agreement on inclusion was reached by consensus’. Unfortunately, we can’t include a number for the % agreement of the 20% screened in duplicate as the software we used (Rayyan) automatically updates any ‘conflicts’ once they’ve been resolved.

6. Similarly in quality appraisal, you note a 20% sample were cross-checked, but if both reviewers independently did this, why were all not checked or what was the outcome of the 20% check, and what happened as a result, especially as you then note, a third reviewer examined discrepancies – was this only on the 20%? Clarity is needed here too.

Thanks again – the reference to 20% was a mistake and we hope we have now brought some clarity to this section (see page 10):- ‘Each study was appraised by two authors independently (KF, GM) and then cross-checked by the same authors to ensure consistency. Any studies where there were scoring discrepancies of more than a grade were referred to another author (SD) for moderation. Studies scoring C or higher were included in the final analysis’. We had no discrepancies of a grade or more and agreed on the study scoring ‘D’ which was excluded at the QA stage and is reported in the Results section.

7. You note the data extraction was carried out by two reviewers – was this independently done and cross-checked or divided equally? Please clarify.

Thank you – we have revised the text in this section to better explain the approach (see page 11) - ‘In a process aligned with thematic synthesis [21] two review authors (KF & GM) worked together to develop codes from the author findings and quote material in the included papers. Using an iterative process the same two authors (KF, GM) then synthesized these codes into descriptive themes (review findings) and reached agreement on the analytical themes following input and discussion from a third author (SD)’.

8. Did the two authors collectively develop the descriptive themes, or independently? please clarify. Similarly, were the GRADE-CERQual assessments done collectively or independently and cross-checked?

See above for how the descriptive themes were developed. The GRADE-CERQual assessment exercise was conducted as follows (see page 11):- ‘KF conducted GRADE-CERQual [22] assessments of the descriptive themes (review findings) and the final grade was reached by consensus following a cross-check of each assessment by GM. Any grade disagreements were referred to SD for moderation’

9. In the results of search section, a sentence should not begin with a numerical digit, that is, if beginning a sentence with a number, the number should be written out in full (e.g., 620 articles, becomes six hundred and twenty…, etc.).

Thank you for pointing this out – we have now written the number out in full

10. I don’t quite understand the inclusion of GRADE-CERQual in Table 2. GRADE-CERQual is applied to discrete findings’ statements, yet here it looks like they are applied to the themes (and unclear if this is the descriptive or analytical theme).

Apologies if this is not clear – in accord with the GRADE-CERQual approach and the method selected (Thematic Synthesis) the discrete findings statements are listed as descriptive themes in column 1 of the table; the contributing studies for each descriptive theme are listed in column 2; the GRADE CERQual assessment (for each descriptive theme) are listed in column 3; supporting quotes for each descriptive theme appear in column 4 and the relevant analytical theme is highlighted in column 5.

What I would suggest is to remove any findings from this Table, and report these in a separate Table with the assessment or certainty. For example, under ‘Importance of screening women early’ there appears to be two separate findings’ statements, but one certainty rating, yet when you read the narrative of the analytical theme ‘Beyond the routine of antenatal screening’ there are certainly more than two discrete findings’ statements identifiable and each of these should be extracted out and subjected to GRADE-CERQual.

Again, apologies if this is not clear….I’m wondering if there is a fault in the table on the copy sent to you. The ‘Importance of screening women early’ should appear in a distinct row (in bold text) alongside the contributing studies, CERQual grading and supporting quotes for this particular descriptive theme, with the relevant analytical theme it belongs to at the end of the row. The next descriptive theme, ‘Screening as a moral imperative’ should appear in the row below with the contributing studies, CERQual grading, supporting quotes and relevant analytical theme at the end….and so on for all the descriptive themes. The table should make it clear (in column 5) that there are:-

- 3 descriptive themes supporting the analytical theme, ‘Beyond the Routine of Antenatal Screening’.

- 5 descriptive themes supporting the analytical theme, ‘Myths, Misunderstandings and Submissiveness’.

- 4 descriptive themes supporting the analytical theme, ‘Fear and uncertainty in the face of adversity’.

- 2 descriptive themes supporting the analytical theme, ‘The importance of familial and organizational support’.

If this is not reflected in the table we can add some text at the beginning of results section to clarify how the descriptive themes inform the analytical themes.

If there is anything else we can do to aid understanding of this table we would be happy to revise.

This goes for the remaining analytical themes and their associated findings. This links to the Appendix, where again it appears the descriptive themes are being subjected to GRADE-CERQual rather than discrete findings. Attention is needed here to adhere to GRADE-CERQual in the assessment of certainty in findings (not themes) and the links between the assessment in the manuscript and that of the evidence profile in the Supp File.

Hopefully our revised text about GRADE-CERQual in the Methods section (see comment 8 above) should make it clear that we applied GRADE-CERQual to the descriptive themes, which, in our view, represent ‘discrete findings’ and an appropriate unit of analysis for GRADE-CERQual assessment. As we understand it “When using CERQual, we assess confidence in each individual review finding. We acknowledge that review findings can be presented in a range of ways (e.g., as themes or theories) as well as at different levels (e.g., descriptive/aggregative and interpretive)” [See link below for details].

Applying GRADE-CERQual to qualitative evidence synthesis findings: introduction to the series | Implementation Science | Full Text

The final excel page on our S2 appendix represents a Summary of Findings (SoF) table in accord with guidance from the GRADE-CERQual development group – see tables 3 and 4 on the link below.

Applying GRADE-CERQual to qualitative evidence synthesis findings—paper 2: how to make an overall CERQual assessment of confidence and create a Summary of Qualitative Findings table | Implementation Science | Full Text

By way of context the GRADE-CERQual assessed descriptive themes (review findings) were used to inform the recently published WHO Guidelines on Sickle Cell Disease in Pregnancy, Childbirth and the Interpregnancy period. [WHO recommendations on the management of sickle-cell disease during pregnancy, childbirth and the interpregnancy period]. The analytical themes presented in this article represent an additional interpretive step in accord with the method used (thematic synthesis).

Thank you for taking the time to review this article. We hope our responses above provide further clarity and address all of the issues raised.

Attachment Submitted filename: Response to Reviewers 08.09.25 KF.docx

# Peer review: Decision Letter 2

**DOI:** 10.1371/journal.pone.0352992.r005

**Authors:** Katibe Tugce Temur, Abiola Olaleye, Abiola Olaleye

11 Dec 2025

PONE-D-25-01416R2

Sickle cell disease and maternity care: A qualitative synthesis of women’s and health providers experiences.

PLOS One

Dear Dr. Finlayson,

Thank you for submitting your manuscript to PLOS ONE. After careful consideration, we feel that it has merit but does not fully meet PLOS ONE’s publication criteria as it currently stands. Therefore, we invite you to submit a revised version of the manuscript that addresses the points raised during the review process.

Please submit your revised manuscript by Jan 25 2026 11:59PM. If you will need more time than this to complete your revisions, please reply to this message or contact the journal office at plosone@plos.org. When you're ready to submit your revision, log on to https://www.editorialmanager.com/pone/ and select the 'Submissions Needing Revision' folder to locate your manuscript file.

Please include the following items when submitting your revised manuscript:

- A rebuttal letter that responds to each point raised by the academic editor and reviewer(s). You should upload this letter as a separate file labeled 'Response to Reviewers'.
- A marked-up copy of your manuscript that highlights changes made to the original version. You should upload this as a separate file labeled 'Revised Manuscript with Track Changes'.
- An unmarked version of your revised paper without tracked changes. You should upload this as a separate file labeled 'Manuscript'.

-->If you would like to make changes to your financial disclosure, please include your updated statement in your cover letter. Guidelines for resubmitting your figure files are available below the reviewer comments at the end of this letter.

If applicable, we recommend that you deposit your laboratory protocols in protocols.io to enhance the reproducibility of your results. Protocols.io assigns your protocol its own identifier (DOI) so that it can be cited independently in the future. For instructions see: https://journals.plos.org/plosone/s/submission-guidelines#loc-laboratory-protocols. Additionally, PLOS ONE offers an option for publishing peer-reviewed Lab Protocol articles, which describe protocols hosted on protocols.io. Read more information on sharing protocols at https://plos.org/protocols?utm_medium=editorial-email&utm_source=authorletters&utm_campaign=protocols.

We look forward to receiving your revised manuscript.

Kind regards,

Abiola Olukayode Olaleye, MBBS, MPH, FWACP

Academic Editor

PLOS One

Journal Requirements:

If the reviewer comments include a recommendation to cite specific previously published works, please review and evaluate these publications to determine whether they are relevant and should be cited. There is no requirement to cite these works unless the editor has indicated otherwise.

Please review your reference list to ensure that it is complete and correct. If you have cited papers that have been retracted, please include the rationale for doing so in the manuscript text, or remove these references and replace them with relevant current references. Any changes to the reference list should be mentioned in the rebuttal letter that accompanies your revised manuscript. If you need to cite a retracted article, indicate the article’s retracted status in the References list and also include a citation and full reference for the retraction notice.

[Note: HTML markup is below. Please do not edit.]

Reviewers' comments:

Reviewer's Responses to Questions

**Comments to the Author**

1. If the authors have adequately addressed your comments raised in a previous round of review and you feel that this manuscript is now acceptable for publication, you may indicate that here to bypass the “Comments to the Author” section, enter your conflict of interest statement in the “Confidential to Editor” section, and submit your "Accept" recommendation.

Reviewer #4: All comments have been addressed

Reviewer #5: (No Response)

Reviewer #6: All comments have been addressed

**********

2. Is the manuscript technically sound, and do the data support the conclusions?

The manuscript must describe a technically sound piece of scientific research with data that supports the conclusions. Experiments must have been conducted rigorously, with appropriate controls, replication, and sample sizes. The conclusions must be drawn appropriately based on the data presented.

Reviewer #4: Yes

Reviewer #5: Yes

Reviewer #6: Partly

**********

3. Has the statistical analysis been performed appropriately and rigorously?

Reviewer #4: N/A

Reviewer #5: N/A

Reviewer #6: I Don't Know

**********

4. Have the authors made all data underlying the findings in their manuscript fully available?

The PLOS Data policy requires authors to make all data underlying the findings described in their manuscript fully available without restriction, with rare exception (please refer to the Data Availability Statement in the manuscript PDF file). The data should be provided as part of the manuscript or its supporting information, or deposited to a public repository. For example, in addition to summary statistics, the data points behind means, medians and variance measures should be available. If there are restrictions on publicly sharing data—e.g. participant privacy or use of data from a third party—those must be specified.

Reviewer #4: (No Response)

Reviewer #5: No

Reviewer #6: Yes

**********

5. Is the manuscript presented in an intelligible fashion and written in standard English?

PLOS ONE does not copyedit accepted manuscripts, so the language in submitted articles must be clear, correct, and unambiguous. Any typographical or grammatical errors should be corrected at revision, so please note any specific errors here.

Reviewer #4: Yes

Reviewer #5: Yes

Reviewer #6: No

**********

6. Review Comments to the Author

Please use the space provided to explain your answers to the questions above. You may also include additional comments for the author, including concerns about dual publication, research ethics, or publication ethics. (Please upload your review as an attachment if it exceeds 20,000 characters)

Reviewer #4: I understand that the manuscript has had three reviewers, and I have read your responses to those comments. Although the journal editor invited me to assess the manuscript as a new reviewer, I have carefully considered the previous reviewers' feedback and the authors' responses as part of my peer review.

I appreciate your efforts in seeking to get this qualitative review on sickle cell disease published. It is crucial to highlight the care and understanding of this disease, particularly in sub-Saharan Africa, where it accounts for approximately 80% of global cases. The article presents a comprehensive and insightful synthesis of qualitative literature affected by this complex and often underrepresented condition.

I commend your rigorous methodological approach and the depth of analysis demonstrated throughout the review. Your work significantly contributes to the existing body of knowledge by illuminating the psychosocial dimensions of sickle cell disease, which are frequently overlooked in clinical discourse. The nuanced understanding presented in the review has important implications for both research and practice, particularly in enhancing person-centred care and informing culturally responsive interventions, especially in LMICs.

I found the review to be both intellectually enriching and practically relevant. It will undoubtedly serve as a valuable resource for healthcare professionals, educators, and policymakers committed to improving the quality of life for individuals living with sickle cell disease.

Once again, thank you for your contribution.

Reviewer #5: I appreciate the authors’ efforts in addressing an important and understudied area within sickle cell disease care. The manuscript has the potential to make a meaningful contribution to the literature. Please find my comments below, which I hope will assist in improving the clarity, rigor, and overall quality of the paper.

Abstract

Methods:

1.I recommend rephrasing the statement “We were looking for…” to a more formal description, such as: “Studies reporting qualitative data from … were included.”

Results

1.Consider including the countries of the included studies in parentheses for added clarity.

Introduction

1.Lines 118 – 120. These statements are not clear. Do you mean the disease considerations/ complications impact views on contraception, etc? Please revise for clarity.

Methods

1.Search strategy: Include the full meaning of CNKI at first mention. There are several other instances where acronyms are not fully described initially, or the full description appears after the acronym has already been used. Please review the manuscript and ensure consistency throughout.

2.Study Selection:

a.Is translating text a standard practice in systematic reviews? If so, please provide a supporting reference. Additionally, acknowledge potential limitations of using Google Translate instead of a human translator in the limitations section. Also, indicate which article was translated in the results section (I assume this was Xavier’s article).

b.Lines 193-194: This statement is unclear. Consider rephrasing

c.Indicate whether the review was restricted to original research articles. I noticed that a book (Dyson SM) was included; I have further comments on this in the Results section.

d.Include a statement about whether studies that included women with SCD or SCT alongside those from the general population were eligible. Some included studies appear to involve participants without SCD or SCT. Also, clarify in the data extraction section how you ensured that only data relevant to the review objectives (i.e., from women with SCD or SCT) were extracted.

3.Quality Appraisal:

a.The references cited do not match the provided bibliography. Reference 21 in the bibliography is Bulgin et al, not Walsh and Downe. Please review the entire manuscript to ensure all in-text citations match the reference list.

b.Please provide reference(s) for ‘A-D’ scoring.

c.Line 226 – 229: Include reference(s) for GRADE scoring

Results

1.Line 239 - 240: This statement is not clear. Perhaps you meant to write “did not fulfil the inclusion criteria.”

2.Include citations in the description of all analytic themes. In its current state, readers must refer back to Table 3 to identify sources, which is not ideal.

3.Dyson SM: It is generally not standard practice to include books in systematic reviews, and I have several concerns about the inclusion. Was the book peer-reviewed? Can you identify the chapters that provided the data used in the review? Also, do you have references to justify the inclusion of a book? If not, consider excluding the book from your results.

4.Table 2: Consider revising the table for clarity. Combine author and year into one column (e.g., Cox and Beauquier, 2014). Replace the title column with study aims, as this provides a clearer sense of the study’s focus.

5.Dyson SM et al. 2007: This study included individuals without SCD or SCT. How did you identify observations specific to your population of interest?

6.Table 3: The quote provided from reference 34 (Tsianikas et al, 2012) was not from an individual with SCD or SCT. The paper says that P5 was not a carrier. This goes back to my initial comment: how did you ensure that the data used to generate the themes in this review were from the population of interest? Please address this and review all the provided quotes to ensure that they were from the population of interest.

7.Table 3: Capitalize the “I” in “intense feelings…”

Discussion

1.The introduction of the discussion frames findings as relevant only to women with SCD, whereas most included studies focused on individuals with SCT. Please revise to reflect implications for both groups.

2.There are several grammatical errors in this section; please proofread carefully.

3.Second paragraph: You note a lack of knowledge among affected individuals, then discuss implications for the general population. This transition feels abrupt—consider reorganizing for better flow.

4.Lines 413–415: This statement is unclear. Additionally, the claim that there are no SCD-specific interventions for healthcare providers is incorrect. See Reich et al., 2024 (Pain Management and Nursing). Please revise for accuracy and clarity.

Reviewer #6: This study is a literature review regarding the view and experiences of women with sickle cell trait (SCT) or sickle cell disease (SCD) and their providers during pregnancy. PRISMA method was used. The authors refer this manuscript addresses topics assigned by the World Health Organization. The authors have addressed previous comments. It is hard to follow because of long paragraphs. There is lack of clarity between the women with sickle cell disease and those with sickle cell trait.

Specific comments:

1.Introduction. 3rd Paragraph is very long. Consider breaking it down to several shorter ones.

2.It is difficult to understand the issues affecting women with SCT. Please make clearer what are differences affecting women who have SCT from those who have SCD.

3.In Table 2, the articles appear to be related to women with SCD or general practitioners. Are any of the studies addressing women with SCT? If so, which ones.

4. Please do not use contractions in the manuscript, such as didn’t. Rather use did not.

5.In the discussion, paragraphs are long. Please divide to make them more readable.

6.Conclusion: Not clear whether there is any conclusion regarding women with SCT.

**********

7. PLOS authors have the option to publish the peer review history of their article (what does this mean?). If published, this will include your full peer review and any attached files.

If you choose “no”, your identity will remain anonymous but your review may still be made public.

**Do you want your identity to be public for this peer review?**  For information about this choice, including consent withdrawal, please see our Privacy Policy.

Reviewer #4: **Yes:** Adeniyi Olanrewaju Adeleye

Reviewer #5: No

Reviewer #6: No

**********

[NOTE: If reviewer comments were submitted as an attachment file, they will be attached to this email and accessible via the submission site. Please log into your account, locate the manuscript record, and check for the action link "View Attachments". If this link does not appear, there are no attachment files.]

To ensure your figures meet our technical requirements, please review our figure guidelines: https://journals.plos.org/plosone/s/figures

You may also use PLOS’s free figure tool, NAAS, to help you prepare publication quality figures: https://journals.plos.org/plosone/s/figures#loc-tools-for-figure-preparation.

NAAS will assess whether your figures meet our technical requirements by comparing each figure against our figure specifications.

Attachment Submitted filename: REVIEWER COMMENTS.docx

# Author comment: Author response to Decision Letter 3

**DOI:** 10.1371/journal.pone.0352992.r006

22 Dec 2025

Response to Reviewers

Many thanks for taking the time to give our article your careful consideration. We appreciate the time taken to thoroughly review our manuscript and believe your comments add strength and clarity to our existing narrative. This rebuttal letter addresses comments raised by reviewers 5 and 6 and our detailed responses to these queries are outlined below (in red font).

Reviewer #4: I understand that the manuscript has had three reviewers, and I have read your responses to those comments. Although the journal editor invited me to assess the manuscript as a new reviewer, I have carefully considered the previous reviewers' feedback and the authors' responses as part of my peer review.

I appreciate your efforts in seeking to get this qualitative review on sickle cell disease published. It is crucial to highlight the care and understanding of this disease, particularly in sub-Saharan Africa, where it accounts for approximately 80% of global cases. The article presents a comprehensive and insightful synthesis of qualitative literature affected by this complex and often underrepresented condition.

I commend your rigorous methodological approach and the depth of analysis demonstrated throughout the review. Your work significantly contributes to the existing body of knowledge by illuminating the psychosocial dimensions of sickle cell disease, which are frequently overlooked in clinical discourse. The nuanced understanding presented in the review has important implications for both research and practice, particularly in enhancing person-centred care and informing culturally responsive interventions, especially in LMICs.

I found the review to be both intellectually enriching and practically relevant. It will undoubtedly serve as a valuable resource for healthcare professionals, educators, and policymakers committed to improving the quality of life for individuals living with sickle cell disease.

Once again, thank you for your contribution.

Reviewer #5: I appreciate the authors’ efforts in addressing an important and understudied area within sickle cell disease care. The manuscript has the potential to make a meaningful contribution to the literature. Please find my comments below, which I hope will assist in improving the clarity, rigor, and overall quality of the paper.

Abstract

Methods:

1.I recommend rephrasing the statement “We were looking for…” to a more formal description, such as: “Studies reporting qualitative data from … were included.”

Agreed – we have altered the text in accord with the reviewer’s suggestion

Results

1.Consider including the countries of the included studies in parentheses for added clarity.

Agreed – we have amended the text and added the relevant countries in brackets

Introduction

1.Lines 118 – 120. These statements are not clear. Do you mean the disease considerations/ complications impact views on contraception, etc? Please revise for clarity.

Noted - we have altered the text to aid clarity, “Due to the risks associated with transferring the sickle cell gene mutation to their children, reproductive decision-making can be a complex process for women with SCD. Their choice of partner as well as views on contraception and antenatal screening all require careful consideration. For some women, in certain contexts, the option to terminate an affected foetus, may lead to particularly difficult and distressing decision-making”.

Methods

1.Search strategy: Include the full meaning of CNKI at first mention. There are several other instances where acronyms are not fully described initially, or the full description appears after the acronym has already been used. Please review the manuscript and ensure consistency throughout.

There is a glossary on page 4 of the manuscript listing all acronyms and their corresponding wording. However, we have also listed the meaning for CKNI in this section for added clarity. We have also checked the manuscript for any other inconsistencies in our use of acronyms.

2.Study Selection:

a.Is translating text a standard practice in systematic reviews? If so, please provide a supporting reference. Additionally, acknowledge potential limitations of using Google Translate instead of a human translator in the limitations section. Also, indicate which article was translated in the results section (I assume this was Xavier’s article).

As is common practice in systematic reviews with a global scope we did not restrict our searches to studies published in English. Google Translate is a useful tool for translating non-English studies and is particularly efficient with Latin based languages. We have added a reference in the methods section to support the use of Google Translate in this regard*. However, on this occasion, all studies (including those from Brazil) were also available in English so we did not need to use the software for this review. We have added a note in the Results section to make this clear, “All included studies were available in English so we did not need to use Google Translate software”.

* Jackson JL, Kuriyama A, Anton A, Choi A, Fournier JP, Geier AK, et al. The Accuracy of Google Translate for Abstracting Data From Non-English-Language Trials for Systematic Reviews. Ann Intern Med. 2019 Nov 5;171(9):677-679. doi: 10.7326/M19-0891. Epub 2019 Jul 30. PMID: 31357212

b.Lines 193-194: This statement is unclear. Consider rephrasing

Apologies for the confusion. This sentence has been changed to read, “KF and GM selected relevant articles for inclusion with adjudication by a third author (SD) in the event of any disagreement”.

c.Indicate whether the review was restricted to original research articles. I noticed that a book (Dyson SM) was included; I have further comments on this in the Results section.

Agreed – We have added a sentence in the Study Selection section stating that, “References for books and book chapters identified from the databases searches were acquired through the University library and assessed for eligibility, i.e. peer reviewed, reporting relevant, empirical, qualitative data”. We have added a further response to your comments regarding book inclusion in the Results section (under point 3 below).

d.Include a statement about whether studies that included women with SCD or SCT alongside those from the general population were eligible. Some included studies appear to involve participants without SCD or SCT. Also, clarify in the data extraction section how you ensured that only data relevant to the review objectives (i.e., from women with SCD or SCT) were extracted.

Agreed – We have added a sentence under the ‘Criteria for Inclusion’ section stating that, “Studies reporting on haemoglobinopathy screening alongside the general population were also included provided there was sufficient data from women diagnosed with SCT”. We have also added a sentence in the ‘Data Extraction and Analysis’ section which reads, “In studies where the author findings included data from participants not relevant to the research question, i.e. participants who did not have SCD or SCT or had thalassemia rather than SCD, we tried to code and extract data from pertinent sections of the text”. In addition, we have inserted another sentence in the ‘Results’ section which reads, “For the six studies focused on screening some of the findings included data from participants relating to thalassemia and some incorporated data from women who had received a negative SCD or SCT screening result. In all of these cases we tried to extract pertinent data from the population of interest by only coding relevant text and ignoring inappropriate quotes”.

3.Quality Appraisal:

a.The references cited do not match the provided bibliography. Reference 21 in the bibliography is Bulgin et al, not Walsh and Downe. Please review the entire manuscript to ensure all in-text citations match the reference list.

Apologies – we have updated this reference and checked the others

b.Please provide reference(s) for ‘A-D’ scoring.

(Revised) Reference [22] outlines the development of the scoring system and reference [26] illustrates its first use. We have also explained how the scoring system is operationalized in the text and in the S2 Appendix.

c.Line 226 – 229: Include reference(s) for GRADE scoring

(Revised) Reference [24] presents a summary of the GRADE-CERQual scoring system and we have added a further citation to this reference as the reviewer suggests.

There are a series of articles which outline each component of the GRADE-CERQual tool and the associated approaches to scoring. [See this link for further details Official guidance for applying GRADE-CERQual :: Grade-cerqual] We can add these additional references if the reviewer would like. However, we feel that ref [24] encapsulates the tool and the scoring system appropriately and provides an introduction to the additional series of papers should a reader wish to explore further.

Results

1.Line 239 - 240: This statement is not clear. Perhaps you meant to write “did not fulfil the inclusion criteria.”

Apologies – yes, this has been altered accordingly

2.Include citations in the description of all analytic themes. In its current state, readers must refer back to Table 3 to identify sources, which is not ideal.

Agreed – we have added relevant citations to the descriptions of analytic themes

3.Dyson SM: It is generally not standard practice to include books in systematic reviews, and I have several concerns about the inclusion. Was the book peer-reviewed? Can you identify the chapters that provided the data used in the review? Also, do you have references to justify the inclusion of a book? If not, consider excluding the book from your results.

As far as we are aware it is common practice to include books and book chapters in reviews of qualitative literature. As outlined in the Cochrane-Campbell Handbook for Qualitative Evidence Synthesis (2023) Cochrane-Campbell Handbook for Qualitative Evidence Synthesis | Cochrane [chapter 5] and noted in Booth’s methodological review on ‘Searching for qualitative research for inclusion in systematic reviews’ (2016)* “qualitative research is published in books as well as journal articles” and it is important to address this issue when searching for relevant data.

The book by Simon Dyson (2005) included in our review was identified in our database searches. It is a peer reviewed book describing an empirical qualitative study and reports on all aspects of the research process, arguably in far greater detail than one might find in a word-limited journal article. We would also suggest that the identification of particular chapters would not be appropriate in this instance since the full study forms the basis for the book. We hope this addresses your concerns in this regard but would be happy to supply further information if required.

*Booth A. Searching for qualitative research for inclusion in systematic reviews: a structured methodological review. Syst Rev. 2016 May 4;5:74. doi: 10.1186/s13643-016-0249-x. PMID: 27145932; PMCID: PMC4855695

4.Table 2: Consider revising the table for clarity. Combine author and year into one column (e.g., Cox and Beauquier, 2014). Replace the title column with study aims, as this provides a clearer sense of the study’s focus.

Agreed – we have combined the author and date into column 1 of table 2 but we have retained the title as we feel this is an important detail. We have added another column (5) outlining the study aims in accord with the reviewer’s suggestion.

5.Dyson SM et al. 2007: This study included individuals without SCD or SCT. How did you identify observations specific to your population of interest?

See response to point 2(d) in the Study Selection section (above) i.e., “For the six studies focused on screening some of the findings included data from participants relating to thalassemia and some incorporated data from women who had received a negative SCD or SCT screening result. In all of these cases we tried to extract pertinent data from the population of interest by only coding relevant text and ignoring inappropriate quotes”.

6.Table 3: The quote provided from reference 34 (Tsianikas et al, 2012) was not from an individual with SCD or SCT. The paper says that P5 was not a carrier. This goes back to my initial comment: how did you ensure that the data used to generate the themes in this review were from the population of interest? Please address this and review all the provided quotes to ensure that they were from the population of interest.

Apologies – we have removed this quote and replaced it with a quote from a participant from the population interest (see revised Table 3). We have also checked all of the additional quotes to ensure they came from relevant participants. In a broader sense, it was difficult to disentangle pertinent data from the findings of several included studies but, as noted previously, we did try to code only those data that reflected findings from our population of interest.

7.Table 3: Capitalize the “I” in “intense feelings…”

Thank you for pointing this out – we have changed this “I” to a capital.

Discussion

1.The introduction of the discussion frames findings as relevant only to women with SCD, whereas most included studies focused on individuals with SCT. Please revise to reflect implications for both groups.

Agreed – we have restructured the ‘Discussion’ section to highlight pertinent findings for women diagnosed with SCT. The third paragraph now includes the following additional text, “For women diagnosed with SCT following antenatal screening appointments our findings illustrate the sense of shock and surprise when an unexpected result is confirmed, especially when the test is presented to them as ‘routine’. Poor understanding of the test and its implications as well as a general lack of knowledge about the genetic origins of the disease highlight notable deficiencies in women’s understanding of SCT and the need for more relevant, understandable, information resources. In addition, our findings suggest that women diagnosed with SCT during antenatal screening would prefer to take the test prior to conception so that reproductive planning and decision-making can occur before they become pregnant. Pre-relationship or pre-marital screening for SCD has been introduced successfully in some European countries (e.g. Greece and Cyprus) though it may not be suitable in all contexts where the identification of carrier status can lead to discrimination or stigmatization [46]”.

2.There are several grammatical errors in this section; please proofread carefully.

Noted – we have checked the Discussion for grammatical errors and altered text accordingly

3.Second paragraph: You note a lack of knowledge among affected individuals, then discuss implications for the general population. This transition feels abrupt—consider reorganizing for better flow.

Noted – we hope the restructured discussion and additional text on women with SCT addresses this concern and improves the flow of the narrative.

4.Lines 413–415: This statement is unclear. Additionally, the claim that there are no SCD-specific interventions for healthcare providers is incorrect. See Reich et al., 2024 (Pain Management and Nursing). Please revise for accuracy and clarity.

Thank you for providing this additional reference. We have amended the text in this section to include the above reference and have added further narrative to aid clarity, “Recent reviews of studies exploring the impact of SCD focused educational interventions for healthcare professionals found that provider understanding of the condition could be improved using a variety of educational initiatives [44,45]. Importantly, the authors also identified studies which highlighted improvements in provider attitudes towards people with SCD [44,45]. Although this is encouraging neither review identified any studies conducted in a maternity context where specific knowledge gaps around screening for SCT and the impact of pregnancy on women with SCD (and vice-versa) remain. This highlights the need for the development of bespoke SCD focused training courses for health professionals working with women (and their families) in reprod

Attachment Submitted filename: Response to Reviewers 22.12.25.docx

# Peer review: Decision Letter 3

**DOI:** 10.1371/journal.pone.0352992.r007

**Authors:** Katibe Tugce Temur, Abiola Olaleye, Abiola Olaleye, Abiola Olaleye

24 Mar 2026

PONE-D-25-01416R3

Sickle cell disease and maternity care: A qualitative synthesis of women’s and health providers experiences.

PLOS One

Dear Dr. Finlayson,

Thank you for submitting your manuscript to PLOS ONE. After careful consideration, we feel that it has merit but does not fully meet PLOS ONE’s publication criteria as it currently stands. Therefore, we invite you to submit a revised version of the manuscript that addresses the points raised during the review process.

Thank you for providing the revised article and the accompanying details for review.  Overall, most of the previously raised comments appear to have been addressed appropriately. However, two major concerns remain.

First, the language used throughout the manuscript still requires substantial improvement to meet the standards of scholarly writing. In several sections, the authors use first-person expressions such as “we” and “our,” which are generally inappropriate for a scientific manuscript unless specifically permitted by the journal’s style guide. The text should be revised to adopt a more formal, objective, and professional academic tone throughout.

Second, the presentation of the qualitative analysis remains insufficiently developed. Although one table has been included to summarise responses and their interpretation, the analysis would benefit from clearer and more comprehensive presentation, such as in tabular, graphical, or other visual formats. At present, the qualitative findings do not yet provide a complete or fully coherent picture of the analytical process and results.

In addition, careful attention should be given to the journal’s formatting and editing requirements. The manuscript should be revised for consistency in font style, font size, and overall presentation.

Please submit your revised manuscript by May 08 2026 11:59PM. If you will need more time than this to complete your revisions, please reply to this message or contact the journal office at plosone@plos.org. When you're ready to submit your revision, log on to https://www.editorialmanager.com/pone/ and select the 'Submissions Needing Revision' folder to locate your manuscript file.

Please include the following items when submitting your revised manuscript:

- A letter that responds to each point raised by the academic editor and reviewer(s). You should upload this letter as a separate file labeled 'Response to Reviewers'.
- A marked-up copy of your manuscript that highlights changes made to the original version. You should upload this as a separate file labeled 'Revised Manuscript with Track Changes'.
- An unmarked version of your revised paper without tracked changes. You should upload this as a separate file labeled 'Manuscript'.

If you would like to make changes to your financial disclosure, please include your updated statement in your cover letter. Guidelines for resubmitting your figure files are available below the reviewer comments at the end of this letter.

If applicable, we recommend that you deposit your laboratory protocols in protocols.io to enhance the reproducibility of your results. Protocols.io assigns your protocol its own identifier (DOI) so that it can be cited independently in the future. For instructions see: https://journals.plos.org/plosone/s/submission-guidelines#loc-laboratory-protocols. Additionally, PLOS ONE offers an option for publishing peer-reviewed Lab Protocol articles, which describe protocols hosted on protocols.io. Read more information on sharing protocols at https://plos.org/protocols?utm_medium=editorial-email&utm_source=authorletters&utm_campaign=protocols.

As the corresponding author, your ORCID iD is verified in the submission system and will appear in the published article. PLOS supports the use of ORCID, and we encourage all coauthors to register for an ORCID iD and use it as well. Please encourage your coauthors to verify their ORCID iD within the submission system before final acceptance, as unverified ORCID iDs will not appear in the published article. *Only*  the individual author can complete the verification step; PLOS staff *cannot*  verify ORCID iDs on behalf of authors.

We look forward to receiving your revised manuscript.

Kind regards,

Abiola Olukayode Olaleye, MBBS, MPH, FWACP

Academic Editor

PLOS One

Journal Requirements:

1. If the reviewer comments include a recommendation to cite specific previously published works, please review and evaluate these publications to determine whether they are relevant and should be cited. There is no requirement to cite these works unless the editor has indicated otherwise.

2. Please review your reference list to ensure that it is complete and correct. If you have cited papers that have been retracted, please include the rationale for doing so in the manuscript text, or remove these references and replace them with relevant current references. Any changes to the reference list should be mentioned in the rebuttal letter that accompanies your revised manuscript. If you need to cite a retracted article, indicate the article’s retracted status in the References list and also include a citation and full reference for the retraction notice.

[Note: HTML markup is below. Please do not edit.]

Reviewers' comments:

Reviewer's Responses to Questions

**Comments to the Author**

1. If the authors have adequately addressed your comments raised in a previous round of review and you feel that this manuscript is now acceptable for publication, you may indicate that here to bypass the “Comments to the Author” section, enter your conflict of interest statement in the “Confidential to Editor” section, and submit your "Accept" recommendation.

Reviewer #5: All comments have been addressed

Reviewer #7: All comments have been addressed

**********

2. Is the manuscript technically sound, and do the data support the conclusions?

The manuscript must describe a technically sound piece of scientific research with data that supports the conclusions. Experiments must have been conducted rigorously, with appropriate controls, replication, and sample sizes. The conclusions must be drawn appropriately based on the data presented.

Reviewer #5: Yes

Reviewer #7: Yes

**********

3. Has the statistical analysis been performed appropriately and rigorously?

Reviewer #5: N/A

Reviewer #7: Yes

**********

4. Have the authors made all data underlying the findings in their manuscript fully available?

The PLOS Data policy requires authors to make all data underlying the findings described in their manuscript fully available without restriction, with rare exception (please refer to the Data Availability Statement in the manuscript PDF file). The data should be provided as part of the manuscript or its supporting information, or deposited to a public repository. For example, in addition to summary statistics, the data points behind means, medians and variance measures should be available. If there are restrictions on publicly sharing data—e.g. participant privacy or use of data from a third party—those must be specified.

Reviewer #5: Yes

Reviewer #7: Yes

**********

5. Is the manuscript presented in an intelligible fashion and written in standard English?

PLOS ONE does not copyedit accepted manuscripts, so the language in submitted articles must be clear, correct, and unambiguous. Any typographical or grammatical errors should be corrected at revision, so please note any specific errors here.

Reviewer #5: Yes

Reviewer #7: Yes

**********

6. Review Comments to the Author

Please use the space provided to explain your answers to the questions above. You may also include additional comments for the author, including concerns about dual publication, research ethics, or publication ethics. (Please upload your review as an attachment if it exceeds 20,000 characters)

Reviewer #5: I thank the authors for addressing all the comments. I believe this is very important and needed work, and I thank them once again for taking the initiative to do it.

Reviewer #7: Thank you for sharing all the relevant details for the review.

In this article there seems to be 2 major lackings, one is that the language is very unprofessional, refereing to self and using the terms we, our, etc is not the appropriate language for scientific article.

Secondly, the qualitative analysis should be presented in a tabular or graphical or pictorial form to make sense of the analysis and the process is clear. Although they have included one table representing different responses and analysis but something is missing, it doesnt give a completed sense.

All the other comments are well adjusted and revised appropriately.

Make sure to follow the guidelines for formating and editing os the text and similar font and size should be used.

**********

7. PLOS authors have the option to publish the peer review history of their article (what does this mean?). If published, this will include your full peer review and any attached files.

If you choose “no”, your identity will remain anonymous but your review may still be made public.

**Do you want your identity to be public for this peer review?**  For information about this choice, including consent withdrawal, please see our Privacy Policy.

Reviewer #5: No

Reviewer #7: **Yes:** Fatima Afsar

**********

[NOTE: If reviewer comments were submitted as an attachment file, they will be attached to this email and accessible via the submission site. Please log into your account, locate the manuscript record, and check for the action link "View Attachments". If this link does not appear, there are no attachment files.]

To ensure your figures meet our technical requirements, please review our figure guidelines: https://journals.plos.org/plosone/s/figures

You may also use PLOS’s free figure tool, NAAS, to help you prepare publication quality figures: https://journals.plos.org/plosone/s/figures#loc-tools-for-figure-preparation.

NAAS will assess whether your figures meet our technical requirements by comparing each figure against our figure specifications.

# Author comment: Author response to Decision Letter 4

**DOI:** 10.1371/journal.pone.0352992.r008

31 Mar 2026

Response to Reviewers

Many thanks for taking the time to give this article your careful consideration. We appreciate the time taken to review our manuscript and welcome your comments and suggestions. This rebuttal letter addresses comments raised by reviewer 7 and endorsed by the academic editor. Our responses to these queries are outlined below (in red font).

Reviewer #5: I thank the authors for addressing all the comments. I believe this is very important and needed work, and I thank them once again for taking the initiative to do it.

Thank you…much appreciated

Reviewer #7: Thank you for sharing all the relevant details for the review.

In this article there seems to be 2 major lackings, one is that the language is very unprofessional, refereing to self and using the terms we, our, etc is not the appropriate language for scientific article.

We acknowledge that the language is presented in a more informal style and appreciate that this may not appeal to all reviewers and readers. In accord with the reviewer’s suggestion, we have therefore altered all first-person references (‘we’, ‘our’, etc; ) to the more formal passive style, more common in academic literature.

Secondly, the qualitative analysis should be presented in a tabular or graphical or pictorial form to make sense of the analysis and the process is clear. Although they have included one table representing different responses and analysis but something is missing, it doesnt give a completed sense.

Thank you for bringing this to our attention. As the reviewer indicates we have presented a table (table 3) highlighting the descriptive themes (along with supporting quotes) and the analytical themes, broadly in accord with the methodological requirements associated with thematic analysis. Full details of the data extraction, coding and thematic development are included in the S2 appendix where colour coding was used to map the coded data to the emerging themes. This provides transparency and should allow a reader to follow our analysis in a clear and methodical manner. However, to aid clarity we have also added brief sections of text at the beginning of each analytical theme (in the Results section) highlighting which descriptive themes contribute to each analytical theme. We trust this addresses the reviewers’ comments but if there is anything else we can add to help with understanding please let us know.

All the other comments are well adjusted and revised appropriately. Thank you

Make sure to follow the guidelines for formating and editing os the text and similar font and size should be used.

We have tried to follow the journal formatting and editing guidelines throughout.

Thanks again for taking the time to review this article. We hope our responses above provide further clarity and address the issues raised.

Attachment Submitted filename: Response to Reviewers 31.03.26 Revised.docx

# Peer review: Decision Letter 4

**DOI:** 10.1371/journal.pone.0352992.r009

**Authors:** Katibe Tugce Temur, Abiola Olaleye, Abiola Olaleye, Abiola Olaleye, Emma Campbell

18 Jun 2026

Sickle cell disease and maternity care: A qualitative synthesis of women’s and health providers experiences.

PONE-D-25-01416R4

Dear Dr. Finlayson,

We’re pleased to inform you that your manuscript has been judged scientifically suitable for publication and will be formally accepted for publication once it meets all outstanding technical requirements.

Within one week, you’ll receive an e-mail detailing the required amendments. When these have been addressed, you’ll receive a formal acceptance letter and your manuscript will be scheduled for publication.

An invoice will be generated when your article is formally accepted. Please note, if your institution has a publishing partnership with PLOS and your article meets the relevant criteria, all or part of your publication costs will be covered. Please make sure your user information is up-to-date by logging into Editorial Manager at Editorial Manager® and clicking the ‘Update My Information' link at the top of the page. For questions related to billing, please contact billing support.

If your institution or institutions have a press office, please notify them about your upcoming paper to help maximize its impact. If they’ll be preparing press materials, please inform our press team as soon as possible -- no later than 48 hours after receiving the formal acceptance. Your manuscript will remain under strict press embargo until 2 pm Eastern Time on the date of publication. For more information, please contact onepress@plos.org.

Kind regards,

Emma Campbell, Ph.D

Staff Editor

PLOS One

Additional Editor Comments (optional):

Reviewers' comments:

Reviewer's Responses to Questions

**Comments to the Author**

1. If the authors have adequately addressed your comments raised in a previous round of review and you feel that this manuscript is now acceptable for publication, you may indicate that here to bypass the “Comments to the Author” section, enter your conflict of interest statement in the “Confidential to Editor” section, and submit your "Accept" recommendation.

Reviewer #7: All comments have been addressed

**********

2. Is the manuscript technically sound, and do the data support the conclusions?

The manuscript must describe a technically sound piece of scientific research with data that supports the conclusions. Experiments must have been conducted rigorously, with appropriate controls, replication, and sample sizes. The conclusions must be drawn appropriately based on the data presented.

Reviewer #7: Yes

**********

3. Has the statistical analysis been performed appropriately and rigorously?

Reviewer #7: Yes

**********

4. Have the authors made all data underlying the findings in their manuscript fully available?

The PLOS Data policy requires authors to make all data underlying the findings described in their manuscript fully available without restriction, with rare exception (please refer to the Data Availability Statement in the manuscript PDF file). The data should be provided as part of the manuscript or its supporting information, or deposited to a public repository. For example, in addition to summary statistics, the data points behind means, medians and variance measures should be available. If there are restrictions on publicly sharing data—e.g. participant privacy or use of data from a third party—those must be specified.

Reviewer #7: Yes

**********

5. Is the manuscript presented in an intelligible fashion and written in standard English?

PLOS ONE does not copyedit accepted manuscripts, so the language in submitted articles must be clear, correct, and unambiguous. Any typographical or grammatical errors should be corrected at revision, so please note any specific errors here.

Reviewer #7: No

**********

6. Review Comments to the Author

Please use the space provided to explain your answers to the questions above. You may also include additional comments for the author, including concerns about dual publication, research ethics, or publication ethics. (Please upload your review as an attachment if it exceeds 20,000 characters)

Reviewer #7: no comments. The article is now good to go. I have noticed that the author has incorporated the language issues and also the qualitative analsysis is represented in a better way.

**********

7. PLOS authors have the option to publish the peer review history of their article (what does this mean?). If published, this will include your full peer review and any attached files.

If you choose “no”, your identity will remain anonymous but your review may still be made public.

**Do you want your identity to be public for this peer review?**  For information about this choice, including consent withdrawal, please see our Privacy Policy.

Reviewer #7: **Yes:** Fatima Afsar

**********

# Editor report: Acceptance letter

**DOI:** 10.1371/journal.pone.0352992.r010

**Authors:** Katibe Tugce Temur, Abiola Olaleye, Abiola Olaleye, Abiola Olaleye, Emma Campbell

PONE-D-25-01416R4

PLOS One

Dear Dr. Finlayson,

I'm pleased to inform you that your manuscript has been deemed suitable for publication in PLOS One. Congratulations! Your manuscript is now being handed over to our production team.

At this stage, our production department will prepare your paper for publication. This includes ensuring the following:

* All references, tables, and figures are properly cited

* All relevant supporting information is included in the manuscript submission,

* There are no issues that prevent the paper from being properly typeset

You will receive further instructions from the production team, including instructions on how to review your proof when it is ready. Please keep in mind that we are working through a large volume of accepted articles, so please give us a few days to review your paper and let you know the next and final steps.

Lastly, if your institution or institutions have a press office, please let them know about your upcoming paper now to help maximize its impact. If they'll be preparing press materials, please inform our press team within the next 48 hours. Your manuscript will remain under strict press embargo until 2 pm Eastern Time on the date of publication. For more information, please contact onepress@plos.org.

You will receive an invoice from PLOS for your publication fee after your manuscript has reached the completed accept phase. If you receive an email requesting payment before acceptance or for any other service, this may be a phishing scheme. Learn how to identify phishing emails and protect your accounts at https://explore.plos.org/phishing.

If we can help with anything else, please email us at customercare@plos.org.

Thank you for submitting your work to PLOS ONE and supporting open access.

Kind regards,

PLOS ONE Editorial Office Staff

on behalf of

Dr Emma Campbell

Staff Editor

PLOS One
